# Using the Interplay of Particle and Substrate Roughness to Modulate Particle Arrangements in Non-Close-Packed Colloidal Arrays

**DOI:** 10.1021/acsomega.6c04722

**Published:** 2026-10-01

**Authors:** Melike Barak, Florian Wolf, Daniel Nachtsheim, Christina Graf

**Affiliations:** † Department of Chemical Engineering and Biotechnology, 38968 Darmstadt University of Applied Sciences, Stephanstr. 7, 64295 Darmstadt, Germany; ‡ Eduard-Zintl-Institute of Inorganic and Physical Chemistry, Technical University of Darmstadt, Peter-Grünberg-Str. 12, 64287 Darmstadt, Germany; § EUt+ Institute of Nanomaterials and Nanotechnologies, EUTINN, European University of Technology, European Union, https://www.univtech.eu/eutinn

## Abstract

Arranging colloidal particles on surfaces with tunable interparticle distances, like in non-close-packed structures (NCPS), is important for many applications. However, controlling their formation remains challenging. Although particle and substrate roughness, along with electrostatic repulsion, affect colloidal interactions, their combined effect on colloidal arrangement remains poorly understood. Therefore, model silica particles with smooth, medium-rough, and rough, virus-like morphologies were synthesized. Virus-like particle synthesis was optimized for controlled roughness (*R*_q_ = 1.4–5.1 nm) and low polydispersity (∼5%). Amine-functionalized particles dispersed in water and aqueous LaCl_3_ solutions were transferred onto gold substrates of two different roughnesses to form NCPS. Only the two roughest particle types (*R*_q_ = 4.3 and 5.0 nm) formed three-dimensional aggregates on rough substrates, likely due to pre-aggregation in dispersion, which could be driven by roughness-mediated mechanical interlocking. By varying ionic strength, the effect of electrostatic repulsion on interparticle distances of differently rough particles (*R*_q_ = 0.3–2.1 nm) is investigated. The rougher the particles, the less an increasing ionic strength reduces interparticle distances. At low ionic strengths, interparticle distances of rough particles (*R*_q_ = 2.1 nm) are independent of ionic strength. Smoother substrates promote lateral mobility for all particles, reducing interparticle distances more strongly for smooth particles. However, aggregation of particles with *R*_q_ = 2.1 nm remains almost the same on both substrates (*R*_q_ = 0.57 and 1.62 nm), while it increases significantly for smoother particles, suggesting that roughness-induced frictional forces are more important at short distances. These findings reveal that adjusting surface roughness can facilitate controlled particle arrangements.

## Graphical abstract

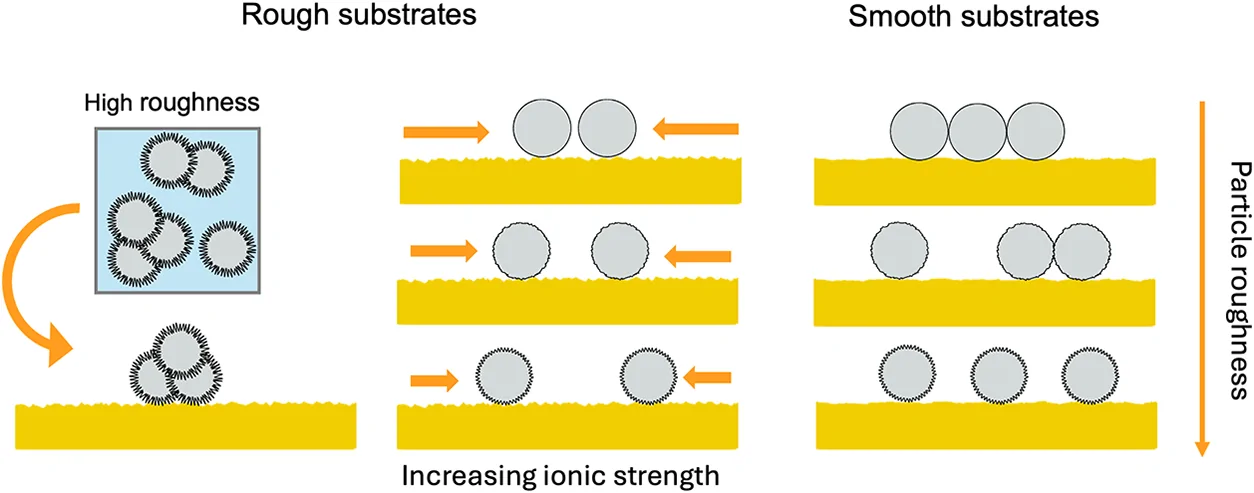

## 1 Introduction

The ability to tailor ordered two-dimensional colloidal arrangements beyond close packing has opened new possibilities for designing functional materials. While hexagonally ordered two-dimensional close-packed colloidal arrays, typically assembled from nearly monodisperse colloidal particles, have been extensively investigated because of their high packing density and long-range periodicity,^1–4^ NCPS often offer greater flexibility over particle arrangement and enhanced surface accessibility. These characteristics can be useful for tailoring properties in several applications, including photonics,^5,6^ plasmonics,^4,7^ biosensors,^8^ superhydrophobic surfaces,^9^ and surface-enhanced Raman spectroscopy.^10,11^ For these applications, on the one hand, precise control of their structural features, such as periodicity, interparticle distance, and particle arrangement, is essential.^12^ On the other hand, quick and straightforward strategies are required to prepare such structures efficiently.

Our group developed a simple colloidal self-assembly method to prepare NCPS on gold substrates, monitored by quartz crystal microbalance with dissipation (QCM-D).^13^ Silica particles with two distinct surface morphologies were amino-functionalized, resulting in a strongly positive zeta potential and enabling binding to a gold surface. Through electrostatic repulsion, NCPS were formed on gold substrates with a low roughness (root-mean-square (RMS) roughness (*R*_q_) = 0.7 nm). Capillary forces strongly influenced the motion of smooth particles during drying, leading to partial aggregation on the substrate. Rough particles were less influenced by these forces due to friction between the particles and the substrate, leading to significantly less aggregation and extended, non-close-packed ordered arrays. This study demonstrates that surface roughness is, in principle, a critical parameter in controlling particle aggregation and interparticle distances. However, this study was limited to particles with only two distinct, relatively low surface roughness values. Additionally, the role of the substrate roughness was not investigated, and the interaction potential between the particles was not tuned. These limitations highlight the need for a more thorough examination of the effects of roughness on particle-particle as well as particle-substrate interactions. This investigation is crucial for understanding the potential of these effects to control two-dimensional colloidal arrays.

Surface roughness is known to critically influence colloidal interactions,^14,15^ including phenomena such as adhesion,^16,17^ adsorption, wettability,^18,19^ and self-assembly.^20,21^ While the Derjaguin-Landau-Verwey-Overbeek (DLVO) theory describes colloidal interactions at larger separations, it fails at distances below a few nanometers, where non-DLVO forces, including steric, capillary, frictional, and hydration forces, become significant.^22^ Capillary forces are particularly sensitive to surface roughness, as asperity distribution and height strongly influence the formation of liquid menisci,^23^ thereby modulating effective particle–particle and particle–substrate interactions. In this context, Rabinovich et al. developed a theoretical model showing that nanoscale roughness reduces capillary adhesion between a solid sphere and a planar surface by limiting meniscus formation at low humidity due to surface asperities. The authors confirmed their theoretical model by using AFM measurements with glass microspheres on substrates with roughnesses ranging from 0.2 to 3 nm.^17^ Notably, the authors found that increasing the roughness by ∼1–2 nm led to a significant reduction of capillary adhesion across multiple surface types. At the monolayer scale, Kato et al. used Langmuir–Blodgett compression isotherms with Blodgett-transfer micrographs to probe monolayers at the air–water interface. Smooth particles formed close-packed monolayers, whereas increasing surface roughness enhanced interlocking and lowered the saturation surface coverage.^15^ Therefore, controlling particle arrangement in non–close-packed monolayers requires a certain roughness which is sufficient to suppress capillary adhesion forces, yet not so high that asperity interlocking dominates. These findings indicate the importance of using model particles and substrates with well-controlled roughness, narrow size distributions, and chemically functionalizable surfaces to enable the formation of well-ordered colloidal monolayers.

Silica particles are promising model systems for colloidal assembly because their size can be tuned over a wide range while maintaining near-monodispersity,^24^ and their surfaces can be modified with functional groups that can prevent particle aggregation.^25^ Furthermore, functionalization enables particles to bind to surfaces, e.g., glass, gold, and biomolecules, and allows the binding strength to be fine-tuned.^26–28^ The van der Waals attraction between silica particles is relatively weak because of the low Hamaker constant of silica.^29^ These properties facilitate the formation of well-ordered self-assembled colloidal monolayers. Different growth mechanisms of colloidal silica, as used in the work of Schmudde et al., provide only limited control of particle roughness.^13^ However, other processes yield silica morphologies with significantly rougher surfaces, which may affect interparticle interactions and assembly behavior. Raspberry-like particles can be produced with controlled root-mean-square roughnesses ranging from 14 to 100 nm;^30^ however, their fabrication typically requires multistep processes. The introduction of some functional groups in the silica network can be used to increase the surface roughness of the particles. Goroncy developed a method for precisely controlling the surface roughness of silica particles within a few nanometers.^31^ In this Stöber-like process, based on a work by Eiden-Assmann et al.,^32^ silica particles with different degrees of surface roughness were obtained by varying the ratio of tetraethoxysilane (TEOS) to the porogen octadecyltrimethoxysilane (ODS). To extend this work, we conducted preliminary experiments using these particles for NCPS preparation. However, the presence of octadecyl chains hinders further surface modification. The removal of these chains by calcination strongly reduces the colloidal stability of these particles, so they cannot be used for preparing structures with well-controlled interparticle distances. Another approach, based on Chen et al., involves functionalizing the particles with thiol groups and, according to the authors, allows precise modification of surface roughness.^33^ Although thiol groups have a high affinity for gold surfaces, achieving a uniform particle shape at higher roughnesses is challenging with this approach. This effect becomes more pronounced, particularly when 3-mercaptopropyltrimethoxysilane (MPTMS) and TEOS are added simultaneously. Cetyltrimethylammonium bromide (CTAB) is a common structure-directing surfactant for the synthesis of mesoporous silica. In water, it tends to form cylindrical micelles, which facilitate the formation of porous silica with tunable pore diameters. The CTAB/TEOS ratio in these processes is an effective parameter for controlling pore formation and templating, particle size, and colloidal stability.^34^ Moreover, CTAB is used to synthesize virus-like silica particles; Wang et al. established this synthesis using CTAB and TEOS via single-micelle epitaxial growth to obtain particles with spiky morphologies.^35^ In this study, mesoporous silica cores are first formed in a cyclohexane/water biphasic system. Upon reactant depletion, silicate oligomers deposit at the particle surface and pore necks, providing nucleation sites for perpendicular spike growth, which can be tuned by varying the reaction time to control both spike length and particle size. However, because spike length and particle size increase concurrently, particle size remains small at lower roughness values, and preparing monodisperse particles using this approach is challenging.^36,37^ Nevertheless, the unique morphology by Wang et al. can serve as a foundation for tailoring particle surface roughness, which plays a critical role in NCPS formation.

To further simplify the preparation of NCPS, we subsequently replaced QCM-D with an orbital shaker.^38^ Using this approach, we showed that the nearest-neighbor distance within the resulting arrays can be readily tuned by varying the ionic strength of the colloidal particle dispersion. This tunability can be further extended by employing specific ion effects and varying the ion valency.^39,40^ For example, using amino-functionalized silica particles deposited on gold substrates in the presence of LaCl_3_, we achieved the lowest aggregation percentage in the NCPS across a wide range of ionic strengths.^40^ Nonetheless, these studies were restricted to a single particle and substrate type. Thus, it was not investigated how roughness effects can tune electrostatic interactions between particles on surfaces and vice versa.

In light of these findings, we conducted a more comprehensive study of the tunability of silica particle roughness on substrates with varying roughness and its impact on NCPS formation by altering ionic strength. By modulating ionic strength, the interparticle distance can be reduced, enabling isolation of surface roughness effects from other parameters controlling particle arrangement. Accordingly, silica particles with different surface roughnesses were synthesized using three different methods, resulting in distinct morphologies: smooth, medium-rough, and rough, virus-like. The synthesis of the virus-like particles was further investigated to control particle size, roughness, and polydispersity. Well-defined model particles with controlled size distributions, low polydispersity, and systematically varied surface roughness were successfully obtained and subsequently functionalized with *N*-(6-aminohexyl)­aminopropyltrimethoxysilane. These amino-functionalized silica particles, dispersed in water and in aqueous LaCl_3_ solutions of varying ionic strength, were deposited onto gold substrates with two different surface roughnesses via colloidal self-assembly. The prepared NCPS were analyzed using field-emission electron microscopy (FESEM) to determine interparticle distances and aggregation percentages. This approach provides a straightforward method for examining and modulating particle-particle and particle-substrate interactions during the formation of NCPS.

## 2 Experimental Section

### 2.1 Materials

Cetyltrimethylammonium bromide (CTAB, ≥98), cyclohexane (99.5%), l-arginine (≥98%), ammonia solution (25% wt. in water), tetraethyl orthosilicate (TEOS, 98%), ethanol (absolute, ≥99.5%), sodium dodecyl sulfate (SDS, >99%), and LaCl_3_·7H_2_O (>99%) were purchased from Sigma-Aldrich. *N*-(6-aminohexyl)­aminopropyltrimethoxysilane (AHAPS, 92%) and NaOH (≥98.0%) were purchased from Fluorochem and Carl Roth, respectively. All chemicals were used as received without further purification. Ultrapure water with a measured resistivity of 18.2 MΩ cm was obtained from a Purelab Flex 2 system (ELGA). The water was immediately flushed with argon for 15 min using an ultrasonic bath (Sonorex Super RK 512 H, Bandelin Electronic), and was then used directly to prepare nanoparticle dispersions.

### 2.2 Synthesis of Smooth Silica Particles (sSP) (*R*_q_ = 0.26 ± 0.04 nm)

Smooth silica particles (sSP) were synthesized based on a method described by Goroncy et al.^31^ First, core particles with a diameter of 25 ± 2 nm were synthesized according to a procedure from Hartlen et al.^41^ Subsequently, the core particles were grown to larger sizes using the method of Reculusa et al.,^42^ enabling the synthesis of particles with a diameter of 144 ± 5 nm in a single step while producing smooth particles. For the particle growth, 0.2 mL of a dispersion of silica core particles without purification in ethanol (10 g/L) was mixed with 10 mL of ethanol and 2.2 mL of ammonia solution at 300 rpm. A mixture of 1 mL TEOS and 4 mL ethanol was prepared and then added to the core particle dispersion using a peristaltic pump at a flow rate of 0.014 mL/min. Once the addition was complete, the mixture was stirred for 16 h. The particles were washed by repeating redispersion in ethanol and centrifugation at 1000*g* three times.

### 2.3 Synthesis of Medium-Rough Silica Particles (mSP) (*R*_q_ = 1 ± 0.3 nm)

Medium-rough silica particles were prepared through a multistep growth process based on a modified Hartlen method^41^ as described in our earlier studies.^41,39^

### 2.4 Synthesis of Virus-like Silica Particles (vSP) (*R*_q_ Ranging from 1.4 ± 0.2 nm to 5.1 ± 0.4 nm)

Virus-like silica particles (vSP) were synthesized using modifications of a procedure proposed by Wang et al.^35^ CTAB (0.055-0.11 mM) and NaOH (0.4-1.6 mM) were added to 50 mL of ultrapure water in a 100 mL round-bottom flask, and the mixture was stirred at 60 °C (50–300 rpm) for 2 h (see Table S1 in the Supporting Info). Afterward, a mixture of 4 mL TEOS and 16 mL cyclohexane was added to the prepared solution and stirred at the same rate for 4 to 6 days. The particles were cleaned by repeated centrifugation and redispersion in water, ethanol, and again in water at 1000*g*. Then, they were dispersed in 50 mL of acetone and refluxed at 50 °C to remove the residual CTAB from the inside of the tubular structure. Subsequently, an additional 50 mL of acetone was added, and the sample was centrifuged at 1000*g*. Finally, the samples were purified by sequentially washing with water followed by ethanol through repeated cycles of redispersion and centrifugation at 1000*g*. Then, the particles were stored in ethanol.

### 2.5 Functionalization of Silica Particles

All particles were modified with *N*-(6-aminohexyl)­aminopropyltrimethoxysilane (AHAPS) under an argon atmosphere, in accordance with the detailed procedure described in ref 43,44

### 2.6 Preparation of NCPS onto Substrates with Different Roughnesses

NCPS were prepared using amino-functionalized particles with different surface roughnesses according to a previously reported method.^38,39^ For studying substrates with a significant surface roughness, particle dispersions (0.1 g/L) in water were deposited onto gold-coated glass substrates (1 cm^2^, 1.5 nm Ti adhesion layer, 200 nm gold thickness, *R*_q_ = 1.62 ± 0.02 nm), purchased from Ssens B.V., Netherlands, using an orbital shaker (VXR Vibrax, IKA). The Au layer had a purity of 99.99 %. To prepare smooth substrates (1 cm^2^), Schott D263 T eco glasses were used and sputtered with a 5 nm chromium layer followed by a 100 nm gold layer of 99.99 % purity using a Leica EM ACE600 high vacuum sputter coater. Prior to the experiments, both substrate types were cleaned using the same procedure: the substrates were cleaned for 10 min using a UV/ozone cleaner (PSD-UV4.Novascan), rinsed with ethanol, and subsequently exposed to a second UV/ozone cleaning for 10 min.

### 2.7 Characterization

Hydrodynamic diameters and electrophoretic mobilities of the silica particles were measured using a Zetasizer Nano ZS (Malvern Instruments Ltd.). The zeta potential was calculated from the measured electrophoretic mobility using the Henry function, f­(κa), where a denotes the particle radius and κ represents the inverse Debye screening length. The value of f­(κa) was determined using the approximation proposed by Swan et al.^45^ Since the measurements were performed at very low ionic strengths (≤ 0.1 mM), the electrophoretic mobilities are also reported as a primary, model-independent experimental parameter. The individual runs yielded highly consistent electrophoretic mobility values, demonstrating good reproducibility within each measurement. The particle size and the particle distribution in the non–close-packed ordered arrays of the silica particles were characterized by field-emission scanning electron microscopy (FESEM, Hitachi SU-5000 Schottky) operated at an accelerating voltage of 5 kV. For each independent experiment, at least six FESEM images were acquired from different regions of the substrate. Depending on the ionic strength, approximately 3000–6000 particles were analyzed in each independent experiment, as higher ionic strengths led to greater surface coverage and consequently allowed more particles to be included in the analysis. The mean particle size of the particles used for NCPS formation was determined by analyzing, on average, 400 particles. The surface roughness of the particles was evaluated by transmission electron microscopy (TEM, Zeiss EM109) at an accelerating voltage of 80 kV. For each synthesis batch, the RMS roughness of ten particles was calculated using a procedure described by Goroncy.^31^ Using FIJI software, the TEM images were converted to binary images by applying a threshold value to pixel intensities via comparing the range of grey values of separated particles and the background, and classifying each pixel as black or white. The outlines of the obtained structures represent the surface profiles of the particles. The distance between each profile coordinate and the center coordinate is calculated to obtain the specific radius *r_i_* of each profile point *i*. Then, the radius deviation *R_i_* is calculated by subtracting the mean radius *r̅* from *r_i_*. These *R_i_* values are then assigned to the corresponding angles, producing a sinusoidal curve along the radial profile. The height profile of this curve superimposes the surface roughness. The resulting curve is then fitted using a Savitzky–Golay filter. The values derived from the Savitzky–Golay fit, *R*_SG_, are subtracted from *R_i_* to obtain the deviation between the measured and the fitted radius deviation *R*_fit_. The standard deviation of *R*_fit_ is the RMS roughness. The substrate roughness was measured using atomic force microscopy (AFM; Cypher, Asylum Research, Oxford Instruments, Santa Barbara, USA) equipped with a PPP FM-AuD10 cantilever (Nanosensors, Neuchâtel, Switzerland). Gwyddion software was used to calculate the RMS roughness. Nearest-neighbor (center-to-center) distances were determined from the FESEM images using the FIJI software with the Simple Analysis 2*D*/3D plugin.

## 3 Results and Discussion

### 3.1 Controlling the Surface Morphology and Size of Silica Particles

Three different methods were used to synthesize silica particles with narrow size distributions, comparable sizes, and a broad range of surface roughness.

#### 3.1.1 Synthesis of Smooth and Medium-Rough Silica Particles

Smooth silica particles (sSP) with a diameter of 144 ± 5 nm and a root mean square roughness (*R*_q_) of 0.26 ± 0.04 nm were prepared by a modified Stöber method according to a method of Goroncy.^31^ The Hartlen method was used to obtain medium-rough silica particles (mSP),^41^ yielding a diameter of 148 ± 6 nm and an *R*_q_ of 1.0 ± 0.3 nm. The differences in the final surface morphology of these particles arise from distinct differences in the condensation mechanisms induced by the solvent used during synthesis.^46^ As reported in earlier studies, silica particles synthesized in ethanol exhibit a smooth surface morphology, whereas those synthesized in water exhibit a rough surface structure. This variation in surface morphology arises from differences in the condensation reactions; specifically, in aqueous media, the irreversible formation of Si–O–Si linkages leads to the development of a rougher morphology.^46^

#### 3.1.2 Synthesis of Rough Virus-like Particles

In addition to the aforementioned syntheses, virus-like silica particles (vSP) were prepared using a modified single-micelle epitaxial growth process described by Wang et al.^35^ During this synthesis process in a cyclohexane/water biphasic system, CTAB is used as a structure-directing template, NaOH as a catalyst, and TEOS as a precursor. Initially, mesoporous silica particles form, and over time, particle size and spike length gradually increase, resulting in a virus-like morphology. Previous studies showed that spike length and particle size increase with the reaction time over 48 h^35^ and 96 h.^37^ However, the resulting particles were smaller than needed in the present work. Hence, we assume that particle growth after these reaction times had not yet been completed; therefore, the reaction time was extended to 4–6 days to obtain larger particles and achieve the desired particle size. The synthesis of sSP and mSP produces structurally well-defined surfaces that are largely unaffected by variations in reaction parameters,^13^ whereas modifying the reaction conditions in the vSP synthesis enables the development of distinct surface morphologies.^35–37^ Accordingly, we optimized the vSP synthesis and investigated how CTAB and NaOH concentrations, as well as the stirring rate, affect spike length and particle size.

##### 3.1.2.1 Influence of NaOH Concentration on the Size and Roughness of vSP

Since pH strongly affects silica formation kinetics,^47^ the impact of NaOH concentration on vSP size and surface roughness was investigated at a constant CTAB concentration and reaction times of 4 and 6 days. The synthesis of vSP-1 with the lowest concentration of NaOH (0.4 mM, pH 9.85) resulted in gel formation, as shown in Figure S2 in the Supporting Information. Figure 1 shows that particle size and surface roughness decrease with increasing NaOH concentration (from pH 10.25 to 10.64) at a CTAB concentration of 0.055 M, as confirmed by FESEM and TEM images. Particle size and polydispersity values, along with all reaction conditions corresponding to the particles displayed in Figures 1 and 2, are provided in Table S1 in the Supporting Information. After a 6-day reaction time, vSP synthesized at a NaOH concentration of 0.8 mM (pH 10.25) reached a particle size of 160 ± 18 nm and a surface roughness of 5.1 ± 0.4 nm (see Figure 1b). However, after increasing the NaOH concentration to 1.2 mM (pH 10.48), both particle size and surface roughness decreased to 154 ± 12 nm and 4.5 ± 0.3 nm, respectively (see Figure 1c). At a NaOH concentration of 1.6 mM (pH 10.64), the particle size and surface roughness were found to be the lowest, with a particle size of 115 ± 17 nm and a surface roughness of 3.4 ± 0.4 nm (see Figure 1d). Experiments with a reaction time of 4 days yield smaller and less rough particles than those with a reaction time of 6 days, following the trend reported by Wang et al.,^35^ who observed an increase in particle size and spike formation at reaction times from 6 to 48 h. The particle size of vSP decreased from 126 ± 8 nm to 109 ± 11 nm after 4 days of reaction when the NaOH concentration was increased from 0.8 mM to 1.2 mM. This was accompanied by a reduction in surface roughness from 4.7 ± 0.4 nm to 3.4 ± 0.3 nm (see Figure 1e,f). vSP prepared at a NaOH concentration of 1.6 mM have a particle size of 101 ± 10 nm and a surface roughness of 1.4 ± 0.2 nm (see Figure 1g). Literature studies have demonstrated that increasing the NaOH concentration in modified Stöber-like syntheses decreases the particle size of silica and organosilica nanoparticles.^48,49^ At high OH^–^ concentrations, the hydrolysis and condensation of TEOS are promoted, causing the nucleation rate to increase relative to particle growth. As a result, more nuclei form, and a smaller average particle size results. Asefa et al. investigated the controlled etching of silica particles produced in a Stöber process at pH values between 10.6 and 12.6 for 1–8 h.^50^ The etching rate increased with the higher hydroxide ion concentration, which causes hydrolysis of siloxanes, resulting in progressively more corrugated particle morphologies, particularly at longer reaction times. Since the pH values in the present study range from 10.25 to 10.64, etching is expected to occur at a slower rate. Spike-like structures on the surface of virus-like particles are expected to be thermodynamically less stable than spherical particles; as a result, the spikes are preferentially etched. This results in a smoother surface morphology with increasing pH and reaction time, rather than an increase in porosity and surface roughness, as is usually observed for silica particles without such spikes.^51,52^

**Figure 1:**
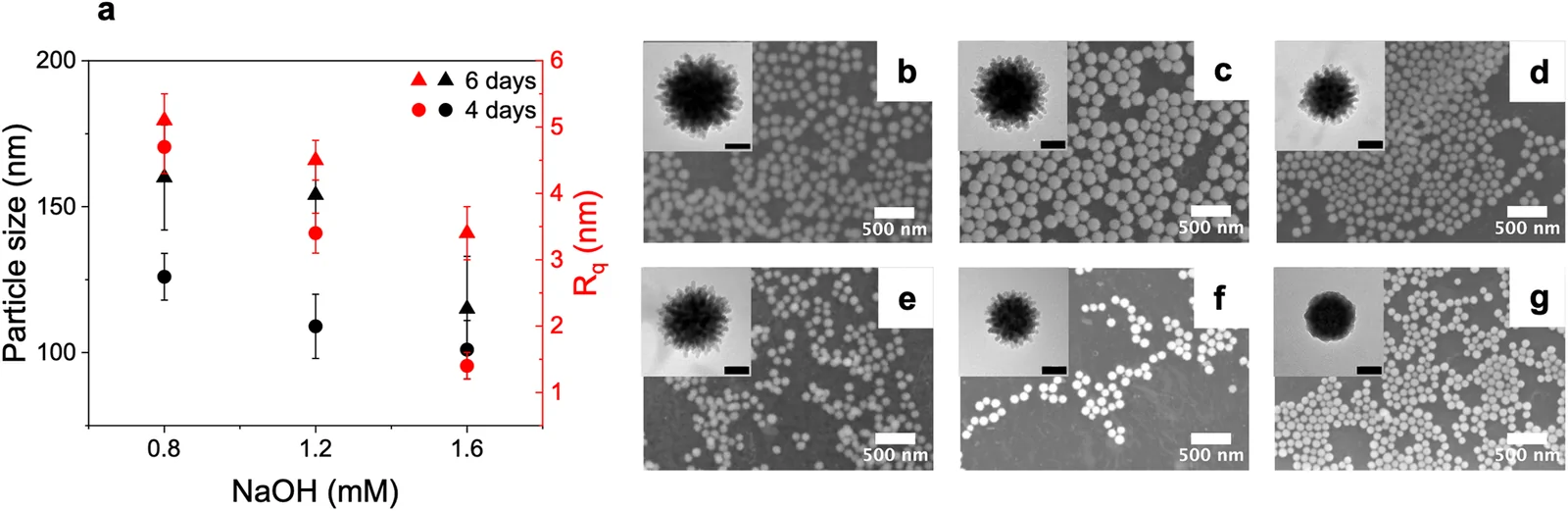
Effect of NaOH concentration on the particle size and roughness: (a) Concentration of NaOH versus particle size and the *R*_q_ of the particles and corresponding FESEM and TEM images (insets) of vSP prepared with a constant CTAB concentration (0.055 M) over 6 days with three different NaOH concentrations: (b) 0.8 mM (vSP-8), (c) 1.2 mM (vSP-5), and (d) 1.6 mM (vSP-6) and over 4 days with three different NaOH concentrations: (e) 0.8 mM (vSP-22), (f) 1.2 mM (vSP-23), and (g) 1.6 mM (vSP-31). All TEM scale bars are 50 nm.

**Figure 2:**
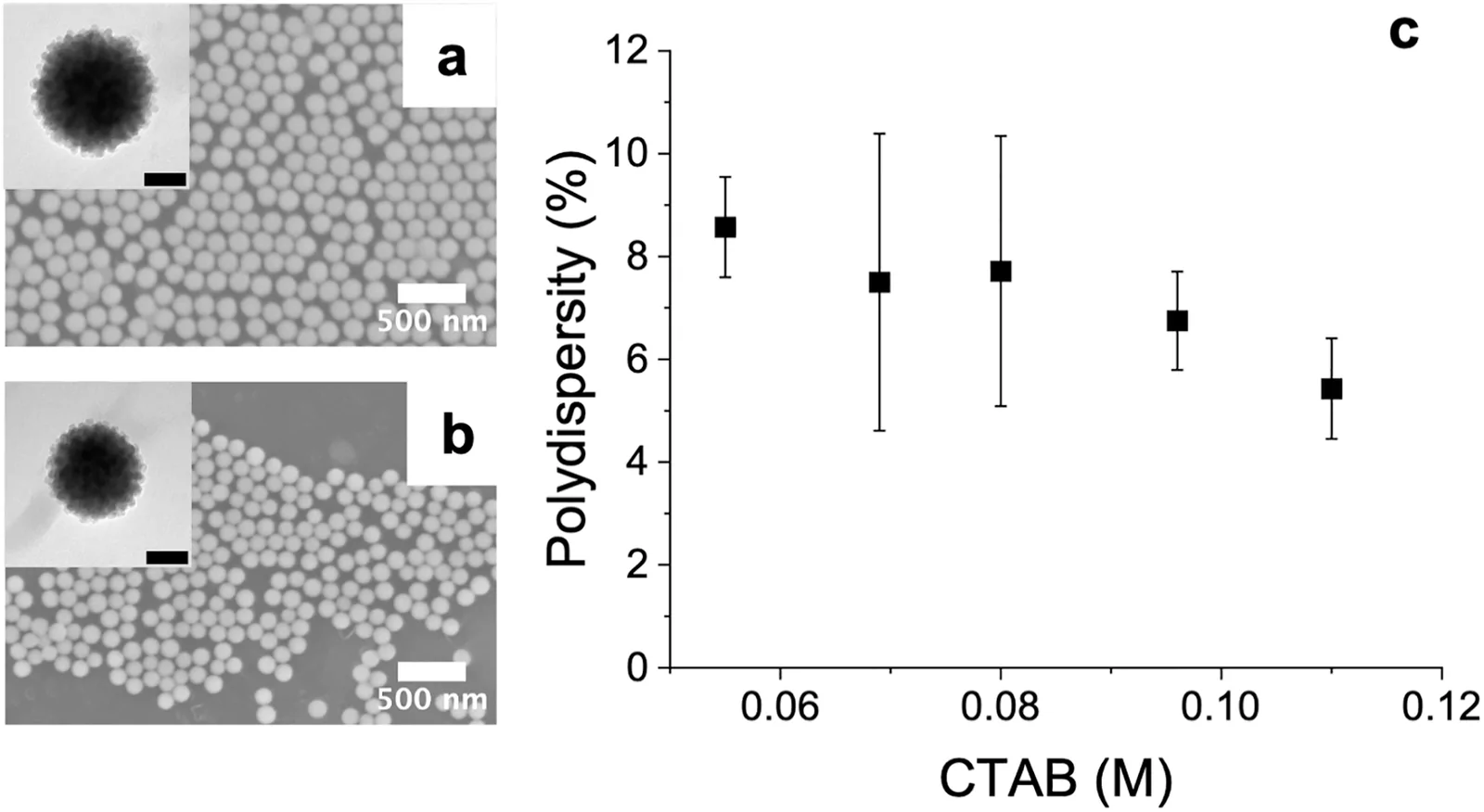
FESEM and TEM images of vSPs prepared with 1.2 mM NaOH and 0.11 M CTAB for reaction times of (a) 6 days (vSP-3) and (b) 4 days (vSP-21), and (c) average polydispersities of vSP synthesized at a constant NaOH concentration (1.2 mM) and stirring rate (300 rpm), as a function of the CTAB concentration. All TEM scale bars are 50 nm.

##### 3.1.2.2 Influence of CTAB on the Size and Roughness of Virus-Like Silica Particles

CTAB is the key reactant that directs the transformation of initially mesoporous silica particles into vSP with their unique morphology. In the work of Wang et al., virus-like particles were obtained at a constant CTAB concentration.^35^ Pinna et al. examined the effect of CTAB concentrations ranging from 0.01 to 0.06 M on spike length and particle diameter. However, the particle polydispersity ranged from 10 % to 20 %.^37^ In the present study, we investigated how CTAB influences polydispersity, aiming to achieve low polydispersity while retaining a spiky virus-like structure. The synthesis of such particles is crucial for preparing non-close-packed structures (NCPS) in subsequent experiments. For this reason, the CTAB concentration range was extended to 0.11 M. The effect of the CTAB concentration and the reaction time on particle size and surface roughness is presented in Table 1. The reaction time was increased to a maximum of 6 days to achieve a particle size comparable to that of the sSP and mSP samples. The results show that the particle size and surface roughness of vSP increase with reaction time. Additionally, an increase in surface roughness was observed with decreasing CTAB concentration. In the 6-day experiments, raising the CTAB concentration from 0.055 to 0.11 M resulted in almost no change in particle size (154 ± 12 nm to 153 ± 7 nm). Meanwhile, the surface roughness decreased from 4.5 ± 0.3 nm to 2.5 ± 0.2 nm, as shown in the FESEM and TEM images (Figure 1c and Figure 2a**)**. Nonetheless, for a 4-day reaction time, increasing the CTAB concentration caused the particle size to rise from 109 ± 11 nm to 121 ± 8 nm, with surface roughness declining from 3.4 ± 0.3 nm to 1.5 ± 0.1 nm, as shown in the corresponding FESEM and TEM images in Figure 1f and Figure 2b. Pinna et al. similarly identified this inverse correlation between particle size and surface roughness.^37^ They suggested that this phenomenon arises from a concentration-dependent switch between core growth and spike formation. High CTAB concentrations promote larger cores with shorter spikes, whereas low CTAB concentrations lead to smaller cores with longer spikes and rougher morphology.^37^ For a reaction time of 4 days, our experimental results are consistent with this observation. Overall, the results of the 4- and 6-day experiments indicate that the particle size varies between batches. Although the reproducibility of the particle size might be further improved through additional synthesis optimization, such studies are beyond the scope of the present work. Nevertheless, the trend of roughness variation across these batches, depending on CTAB concentration, is consistent. Previous studies did not address the effect of CTAB concentration on particle polydispersity. Here, we examined this relationship using the average polydispersity values shown in Figure 2c, calculated from experiments performed at constant NaOH concentration (1.2 mM) and stirring rate (300 rpm). Although the particle polydispersity varies widely, the average value decreases slightly as the CTAB concentration increases. The highest particle uniformity was achieved at a CTAB concentration of 0.11 M, corresponding to an average polydispersity of approximately 5 %. Yamada et al. reported that increasing the surfactant-to-TEOS ratio during the synthesis of mesostructured silica nanoparticles, particularly above 0.13, improves particle dispersibility.^53^ Moreover, the TEM images in ref 53 suggest that polydispersity decreases as the surfactant-to-TEOS ratio increases, consistent with the enhanced dispersibility. In light of this, the higher CTAB concentration may improve the polydispersity of mesostructured silica nanoparticles by suppressing aggregation and promoting a more stable colloidal state. However, further investigation is needed.

**Table 1:** Particle Size Obtained by FESEM Measurements (*D*_SEM_) and *R*_q_ for vSP Prepared with 1.2 mM NaOH and CTAB Concentrations of 0.055 and 0.11 M over 4 and 6 Days

	4 days		6 days	
CTAB (M)	*D*_SEM_ (nm)	*R*_q_ (nm)	*D*_SEM_ (nm)	*R*_q_ (nm)
**0.11**	121 ± 8	1.5 ± 0.1	153 ± 7	2.5 ± 0.2
**0.055**	109 ± 11	3.4 ± 0.3	154 ± 12	4.5 ± 0.3

##### 3.1.2.3 Influence of the Stirring Rate on the Size of Virus-like Particles

The stirring rate, which influences hydrolysis and condensation rates, alters the size and morphology of mesoporous silica particles.^54^ In the present study, the effect of the stirring rate on the particle size and *R*_q_ of vSP prepared at 50, 150, and 300 rpm for 4 days was investigated. The obtained results are shown in Figure 3. No significant change in particle size was observed at stirring rates of 150 and 300 rpm, suggesting that increasing the stirring rate above 150 rpm does not have a measurable influence on particle growth, which can be explained by the rather slow growth process of the particles. However, the particle size of vSP decreased with increasing stirring rate from 50 to 300 rpm from 173 ± 3 nm to 121 ± 8 nm for 0.11 M CTAB and 1.2 mM NaOH, from 188 ± 19 to 126 ± 8 nm for 0.055 M CTAB and 0.8 mM NaOH, and from 170 ± 14 to 109 ± 11 nm for 0.055 M CTAB and 1.2 mM NaOH (see Figure 3a). Pinna et al. investigated the effect of three different stirring rates on the synthesis of virus-shaped silica particles in the presence of *n*-hexane as a cosolvent.^37^ However, their results were inconclusive, as the particles obtained at 250 rpm were larger than those at 150 or 300 rpm. In general, higher stirring rates increase the oil–water interfacial area, accelerating TEOS hydrolysis and nucleation and thereby producing smaller nanoparticles.^55^ Consistent with this, Lv et al. showed that stirring-rate-dependent diffusion controls particle size in mesoporous silica nanoparticle synthesis using TEOS, cetyltrimethylammonium chloride (CTAC), and triethanolamine (TEA), in which lower stirring promoted local monomer supersaturation at the TEOS–water interface and thus the growth of larger particles.^56^ However, no statistically significant effect of the stirring rate on *R*_q_ was observed, as shown in Figure 3b. Nucleation during the synthesis of virus-like particles occurs rapidly; consequently, the stirring rate primarily affects the number of nuclei formed and, ultimately, the particle size. In contrast, spike growth is a slow process that progresses over days (see above) and, hence, is independent of a stirring rate in the order of 150 rpm or more. This suggests that the rate of spike growth is unrelated to the nucleation rate of the core particles. A separation of nucleation and growth of the core particle is supported by the low polydispersity of the final product, indicating that nucleation is completed before particle growth begins. Controlling multiple critical synthesis parameters, e.g., stirring rate, CTAB, and NaOH concentrations, enables us to prepare particles with well-defined sizes, tunable surface roughness, and low polydispersity.

**Figure 3:**
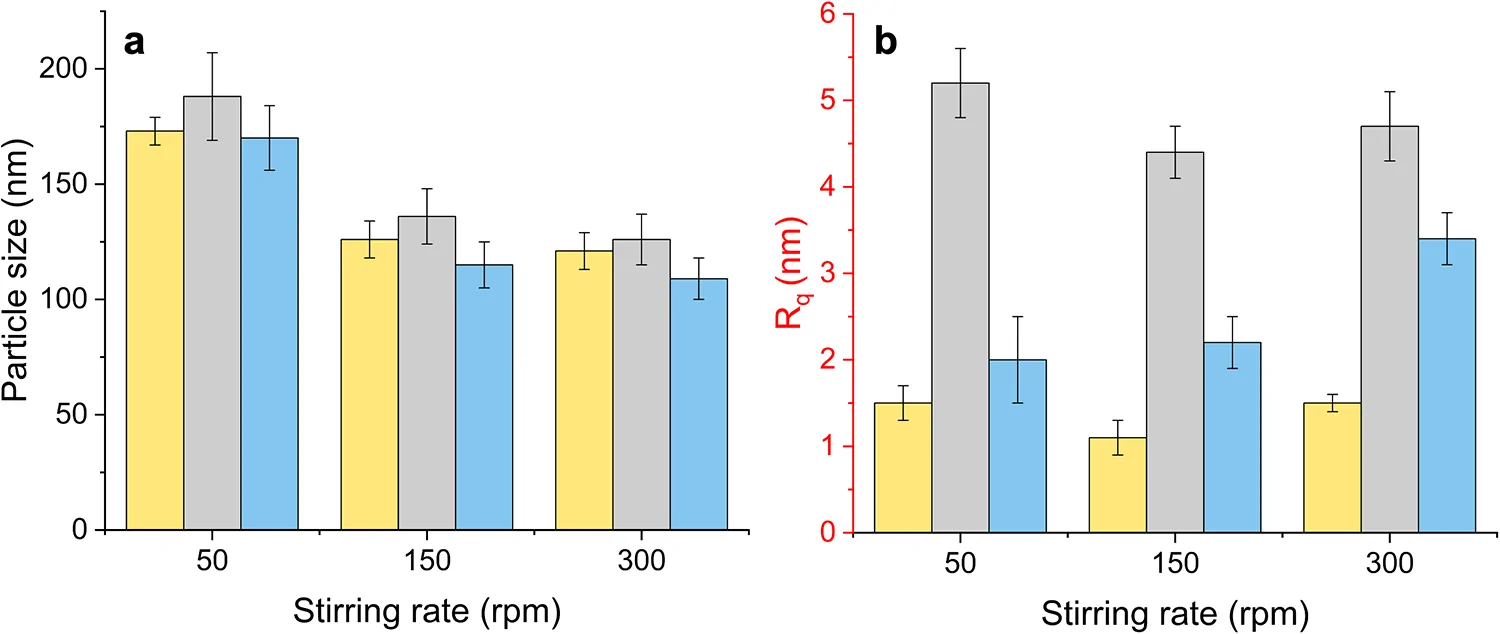
Effect of the stirring rate on the particle size. The histogram shows variations in (a) the particle size distribution and (b) *R*_q_ of vSP synthesized using 0.11 M CTAB and 1.2 mM NaOH (yellow), 0.055 M CTAB and 0.8 mM NaOH (gray), and 0.055 M CTAB and 1.2 mM NaOH (blue), with a reaction time of 4 days at varying stirring rates.

### 3.2 Investigation of Roughness-Controlled Colloidal Self-Assembly

#### 3.2.1 Model Silica Particles for the Formation of Non–Close-Packed Structures (NCPS)

The effects of the roughness of sSP and mSP, as well as virus-like particles (vSP) with three different roughness levels on particle arrangement on the substrate were compared. As a first step, all particles were functionalized with amino groups by adding *N*-(6-aminohexyl)­aminopropyltrimethoxysilane (AHAPS). The surface roughness values of these particles were determined from TEM images (see Figure S1 in the Supporting Information) and are summarized in Table 2. Taking the standard deviation into account, the surface roughness remained almost unchanged after functionalization. The colloidal stability of the silica particles in dispersion was measured using dynamic light scattering (DLS) and electrophoretic light scattering to determine hydrodynamic diameters and zeta potentials (see Table 2). For clearer nomenclature, the amino-functionalized silica particles are designated according to their measured surface roughness after amino functionalization as sSP-0.3, mSP-1.2, vSP-2.1, vSP-4.3, and vSP-5.0.

**Table 2:** Summary of Particle Size Determined by SEM (*D*_SEM_), Surface Roughness Calculated from TEM Micrographs, and Hydrodynamic Diameter (*D*_DLS_) and Zeta Potential Measured in Water for Amino-Functionalized Particles^a^

Properties	sSP-0.3	mSP-1.2	vSP-2.1	vSP-4.3	vSP-5.0
*D*_SEM_ (nm)	147 ± 3	149 ± 3	154 ± 5	154 ± 12	126 ± 8
*R*_q_ (nm)	0.3 ± 0.1	1.2 ± 0.2	2.1 ± 0.1	4.3 ± 0.3	5.0 ± 0.2
*D*_DLS_ (nm)	180 ± 1	171 ± 3	178 ± 2	213 ± 5	181 ± 1
zeta potential (mV)	61 ± 1	85 ± 2	74 ± 2	68 ± 0.6	67 ± 0.3

a The zeta potential data were measured at an ionic strength of 0.01 mM. Electrophoretic mobilities and apparent zeta potential data for ultrapure water are given in the Supporting Information in Table S2.

#### 3.2.2 Arrangement of Amino-Functionalized Silica Particles on Rough Substrates from Water and Aqueous LaCl_3_ Solutions

The amino-functionalized silica particles are deposited from aqueous dispersions onto gold-coated glass substrates with a defined roughness (*R*_q_ = 1.62 ± 0.02 nm) using an orbital shaker, as described in ref 38 Subsequently, the dried NCPS were analyzed using FESEM as shown in Figure 4. While a few aggregates were observed in NCPS prepared with sSP-0.3 (see Figure 4a), well-ordered arrangements were obtained with mSP-1.2 and vSP-2.1. The results of these three samples are discussed in detail below regarding their assembly mechanisms (Figure 4b,c). In contrast, the NCPS prepared from aqueous dispersions of vSP-4.3 and vSP-5.0 exhibited three-dimensional aggregation as shown in Figure 4d,e. The zeta potentials of these two particle types were comparable to those of vSP-2.1. This indicates that all virus-like particles were functionalized with amino groups to a similar extent. However, the hydrodynamic diameters of the samples vSP-4.3 and vSP-5.0 were 213 ± 5 nm and 181 ± 1 nm, respectively. These diameters are significantly larger than those obtained by FESEM. The difference between the diameters obtained by FESEM and DLS is also considerably larger than observed for the other particles (see Table 2). This discrepancy indicates significant aggregation of the highly rough particles in the dispersion and may be attributed to mechanical interlocking between particles driven by their long, spike-like morphology, which enhances physical entanglement and reduces colloidal stability. As a result, three-dimensional aggregates are found on the substrates. Roughness-induced colloidal stability in dispersions has been observed before: Hsu et al. found using raspberry-like silica particles that dispersions of rough particles exhibit shear thickening at much lower volume fractions than smooth colloids due to the interlocking of surface asperities.^57^ By comparing the 3D translational and rotational diffusion coefficients of rough and smooth silica particles in different solvents, Ilhan et al. found that rough particles exhibit purely diffusive behavior in the dilute limit, with diffusion coefficients similar to theoretical values for smooth spheres of the same size.^58^ Accordingly, roughness itself does not significantly increase hydrodynamic diameter. This finding supports the hypothesis that the larger hydrodynamic diameters of vSP-4.3 and vSP-5.0 are due to aggregation. Notably, the difference in relative roughness contrast in ref 58 was substantially larger than in the present case. Pradeep et al. reported that the jamming limit of smooth colloids in dispersion is significantly higher (ϕ*_J_* = 0.64) than that of rough colloids (ϕ*_J_* = 0.56).^59^ They explained this difference by interlocking hindrance, which the authors describe as a form of geometric friction arising from the inability of rough colloids to rotate freely in the dispersion medium.

**Figure 4:**
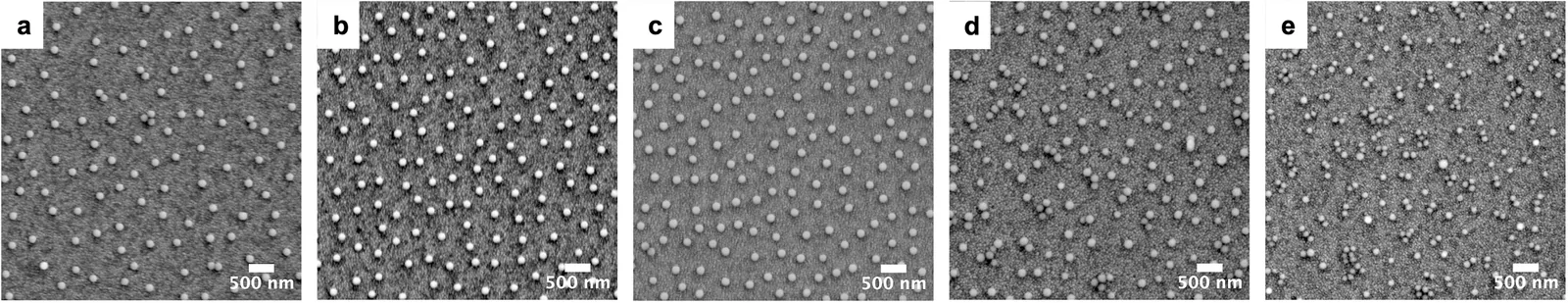
FESEM images of amino-functionalized (a) sSP-0.3, (b) mSP-1.2, (c) vSP-2.1, (d) vSP-4.3, and (e) vSP-5.0 (Note that these particles have a smaller size.) on rough gold substrates. The particles were dispersed in pure water, transferred onto substrates using an orbital shaker, and then dried.

Since the two particle types with the highest surface roughness show significant aggregation in dispersion, only the three particle systems with the lower surface roughnesses (sSP-0.3, mSP-1.2, and vSP-2.1), showing sufficient colloidal stability in dispersion, were used to investigate further the effect of surface roughness on the formation of NCPS. In this study, the ionic strength of the dispersions was employed as a controllable parameter to increase colloidal interactions. With increasing ionic strength, surface charges are screened more effectively, leading to a shorter Debye length and, consequently, reduced electrostatic repulsion. Previously, Schmudde et al. reported a study in which particles from a Hartlen approach similar to sample mSP-1.2 in NaCl dispersions (ionic strengths ranging from 0.05 to 1 mM) were deposited on a gold-coated glass substrate from the same provider as in this study using an orbital shaker, to investigate interparticle distances.^38^ In our present study, minimizing particle aggregation was critical for accurately assessing the effect of surface roughness on particle arrangements. For this purpose, LaCl_3_ was selected, as we recently showed that this trivalent lanthanide chloride resulted in the lowest aggregation percentage in NCPS among all chlorides tested.^40^ In addition, only dispersions with low ionic strength were used to further prevent aggregation. Taking all of these considerations into account, the NCPS were prepared from sSP-0.3, mSP-1.2, and vSP-2.1 in water and LaCl_3_ solutions with ionic strengths ranging from 0.01 to 0.1 mM. The formation of complexes was not observed in LaCl_3_ solutions at concentrations below 0.1 M;^60^ therefore, complete dissociation was assumed for the calculation of the ionic strength. The three types of amino-functionalized particles were colloidally stable in water and LaCl_3_ solutions at various ionic strengths, as indicated by the DLS and zeta potential data (see Table S2 in Supporting Information). In ultrapure water, electrophoretic mobilities could be measured (see Table S2 in the Supporting Information), although it is challenging to obtain a stable and homogeneous electric field under these conditions.^61^ Li et al. investigated the conductivity of ultrapure water under air-saturated and Ar-degassed conditions and showed that the conductivity strongly depends on dissolved CO_2_.^62^ They reported conductivities of 1.33 ± 0.02 μS cm^–1^ for air saturated water and 0.36 ± 0.01 μS cm^–1^ for Ar degassed water. Based on Li et al. and considering our experimental conditions (see Experimental Part), the ionic strength of the ultrapure water was assumed to be ≈0.001 mM. At such a low ionic strength, relatively large uncertainties in the zeta potential values can be expected.^63^ Owing to the large Debye length, the system approaches the Hückel limit. Because of the uncertainty under these conditions, the corresponding zeta potential values are reported as apparent values. For the LaCl_3_ solutions at ionic strengths ranging from 0.01 to 0.1 mM, zeta potentials were calculated using Henry’s equation. In addition, surface morphology may also play a role. A theoretical study showed that nanoscale roughness can affect the electrical double layer and local surface charge.^64^ In this context, changes in ionic strength may influence rough and smooth surfaces differently. However, the underlying mechanism cannot be determined from the zeta potential measurements alone. The interparticle distances in the NCPS were measured using FESEM.

Figure 5 shows the histograms of nearest-neighbor distance distributions in the NCPS obtained using particles with three different surface roughnesses dispersed in water and in aqueous LaCl_3_ solutions with various ionic strengths. In the histograms, two peaks are observed: the left peak corresponds to aggregated particles, while the right peak represents particles with controlled interparticle distances.^38,39^ The average position of the right peak gives the nearest-neighbor distance. Histograms for vSP-2.1, mSP-1.2, and sSP-0.3 at all investigated ionic strengths are shown in Figure S3 in the Supporting Information. Figures 5a–f show the distributions of nearest-neighbor distances at the lowest and highest ionic strengths, highlighting the differences in the behavior of silica particles with different roughness during the formation of NCPS, red lines were drawn in the histograms to mark the average nearest-neighbor distances. To compare ionic strengths, cumulative distribution functions (CDFs) of the nearest-neighbor distances are additionally presented in Figure 5g–i for vSP-2.1, mSP-1.2, and sSP-0.3, respectively. This comparison demonstrates that, with increasing ionic strength, the nearest-neighbor distance decreases, as expected due to reduced electrostatic repulsion between particles, in line with our earlier findings.^38–40^ Strikingly, as particle roughness increases, the influence of ionic strength on the nearest-neighbor distance decreases significantly. Accordingly, vSP-2.1 likely exhibits the greatest interlocking, consistent with its highest surface roughness. In addition, broader distributions of the nearest-neighbor distances were found for NCPS prepared from sSP-0.3. At the highest ionic strength of 0.1 mM, aggregation is quite low in the samples prepared with vSP-2.1 and mSP-1.2. Compared to this, the aggregation peak is notably higher for sSP-0.3 (see Figure S3 in the Supporting Information). In our previous study, the formation of NCPS on relatively smooth gold substrates (*R*_q_ = 0.7 nm) from amino-functionalized smooth and medium-rough silica particles was comparatively investigated using QCM-D measurements, but without the electrostatic repulsion between the particles being systematically tuned.^13^ These particles are similar to the two smoothest types applied in the present work. Please note that the discrepancy in surface roughness values of the silica particles between ref 13 and the present work originates from differences in the roughness calculation methodology. The roughness reported there was greater because the surface profile of the particles was not corrected by subtracting a smoothed sinusoidal curve before calculating the RMS roughness, as described in Section 2.7.^31^ In that study, smoother particles exhibit enhanced aggregation, attributed to increased lateral mobility from weaker particle–substrate interactions, whereas the suppressed aggregation of rougher particles was associated with friction forces. Scherrer et al. reported significant differences in the sliding and rolling behavior of smooth and rough, raspberry-like particles on smooth and rough substrates using the colloidal probe AFM technique.^65^ In that study, the roughness on the particle and substrate surfaces was created by coating of 12 μm silica core with silica nanoparticles of 100 and 300 nm diameter and a glass slide with 50-500 nm diameter of silica, respectively. Although the particle and substrate roughnesses were much higher than in our present study, the ratio of the asperity sizes between the particles and the substrate is comparable. Their results indicate that the probability of interlock events is decreased for smooth particles, thereby increasing their tendency to slide. These findings provide a possible framework for interpreting our observation that particles with smoother surface morphologies yield broader nearest-neighbor distributions during NCPS formation, which may be associated with greater lateral mobility. However, friction forces were not directly measured in the present study. The observed differences in particle arrangement may also arise from surface-morphology-dependent capillary interactions. According to a theory developed by Butt et al., the capillary force between two undeformable, arbitrary shaped, solid surfaces with nanometer-scale roughnesses provide a reduced effective contact area for capillary bridges compared to smooth surfaces. As a result, capillary bridges between rough particles and solid surfaces are smaller than those between smooth particles and surfaces. Moreover, in the former case, the capillary bridges are confined to discrete asperities, rather than a continuous meniscus is formed.^23,66^ Additional support for the effect of roughness on capillary bridging comes from the work of Allard et al., who investigated the influence of surface roughness of fluorescence-labeled raspberry-like microparticles, obtained by adsorbing silica particles ranging from 40 to 250 nm onto the surfaces of core particles. Using confocal microscopy, the authors showed that highly rough particles form capillary bridges mainly between asperities, whereas smoother particles form broader pendular bridges, resulting in weaker particle–particle capillary forces for the rougher particles.^67^

**Figure 5:**
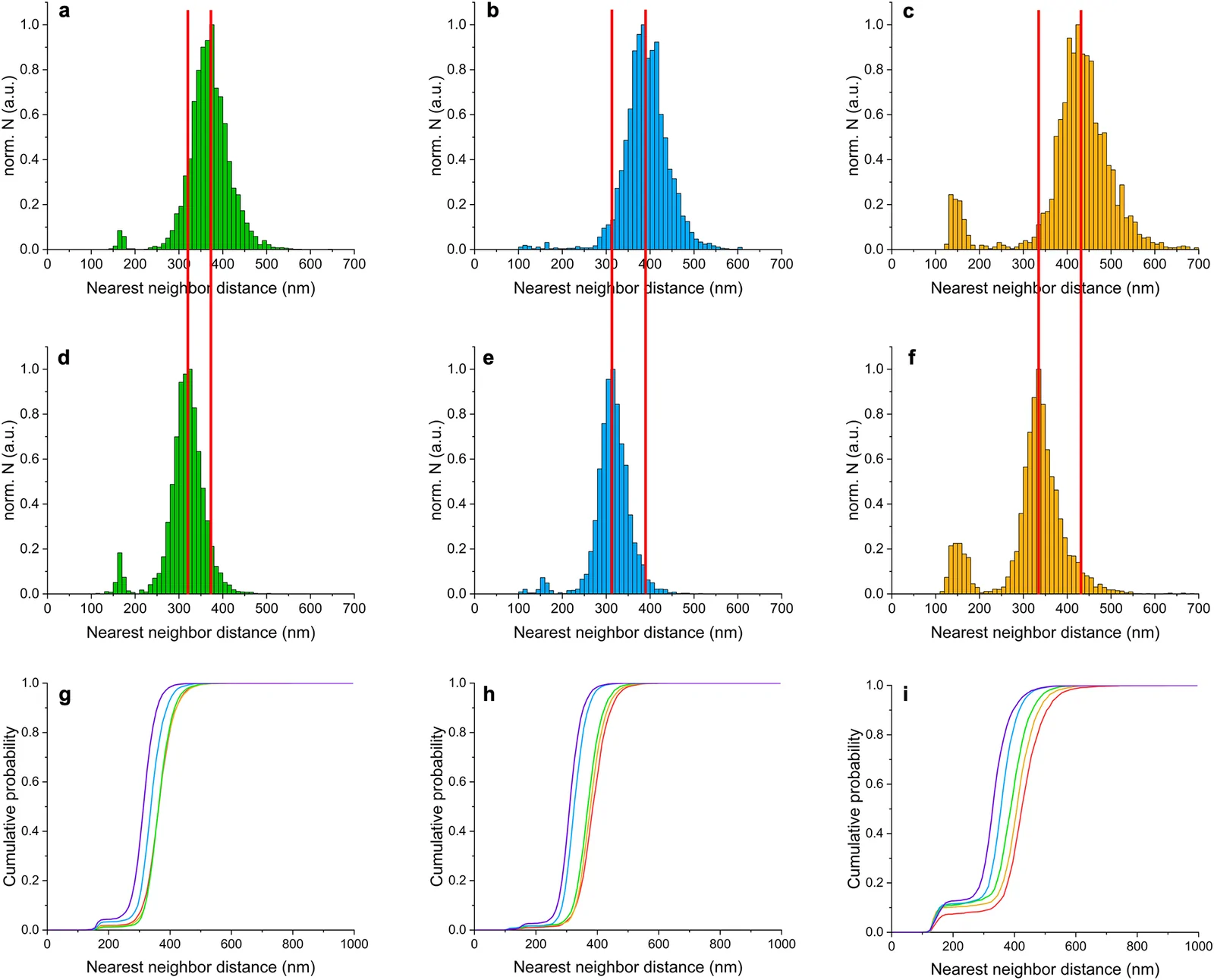
Histograms showing the distribution of the nearest-neighbor distances of NCPS of amino-functionalized silica particles with different roughnesses on gold substrates with *R*_q_ = 1.62 ± 0.02 nm. Nearest-neighbor distance distributions of NCPs prepared from particles dispersed in water: (a) highly rough virus-like silica particles (vSP-2.1, green), (b) medium-rough silica particles (mSP-1.2, blue), and (c) smooth silica particles (sSP-0.3, orange); and from particles dispersed in aqueous 0.1 mM LaCl_3_ solution: (d) vSP-2.1, (e) mSP-1.2, and (f) sSP-0.3. The red lines indicate the average nearest-neighbor distance at the highest and lowest ionic strength values for each class of nanoparticles. The cumulative distribution functions (CDFs), illustrating cumulative probability as a function of nearest-neighbor distance at different ionic strengths, are shown for (g) vSP-2.1, (h) mSP-1.2, and (i) sSP-0.3. The curves represent particles dispersed in water (red) and in aqueous LaCl_3_ solutions with ionic strengths of 0.01 mM (yellow), 0.025 mM (green), 0.05 mM (blue), and 0.1 mM (purple).

In the present study, the greater aggregation observed for smooth particles arises from roughness-dependent differences in particle–particle capillary interactions during drying. Although the NCPS produced by colloidal self-assembly were dried slowly to reduce capillary forces, such interactions could still contribute to the observed aggregation. Conversely, surface roughness may reduce particle–substrate capillary adhesion, potentially increasing particle mobility. However, frictional and interlocking interactions between particles may counteract this effect, as discussed above.

Because these mechanisms were not examined directly in the present study, their respective contributions cannot be quantified. Nevertheless, regarding the theoretical models and experimental studies discussed above, the observed differences in particle arrangement are consistent with a combined effect of roughness-dependent capillary interactions, particle mobility, and frictional or interlocking effects, rather than a single dominant mechanism.

Figure 6 shows variations in the nearest-neighbor distance in NCPS of vSP-2.1, mSP-1.2, and sSP-0.3 as a function of the ionic strength. For each experiment, the mean nearest-neighbor distance was determined by Gaussian fitting of the histogram and then averaged over three independent experiments, excluding aggregated particles. The nearest-neighbor distance decreased with increasing ionic strength for all particle types: sSP-0.3 from 432 ± 10 nm to 339 ± 8 nm, mSP-1.2 from 390 ± 5 nm to 315 ± 6 nm, and vSP-2.1 from 368 ± 3 nm to 319 ± 3 nm. The apparent zeta potential of sSP-0.3 in ultrapure water is much higher compared to the zeta potential of vSP-2.1 (see Table S2) Although, as discussed above zeta potential data at such extremely low ionic strengths are subject to increased experimental uncertainty,^61,63^ this large difference indicates a significantly stronger electrostatic repulsion among the particles in sample sSP-0.3 compared to those in sample vSP-2.1 and, as a result, the largest nearest-neighbor distances are observed for sSP-0.3.

**Figure 6:**
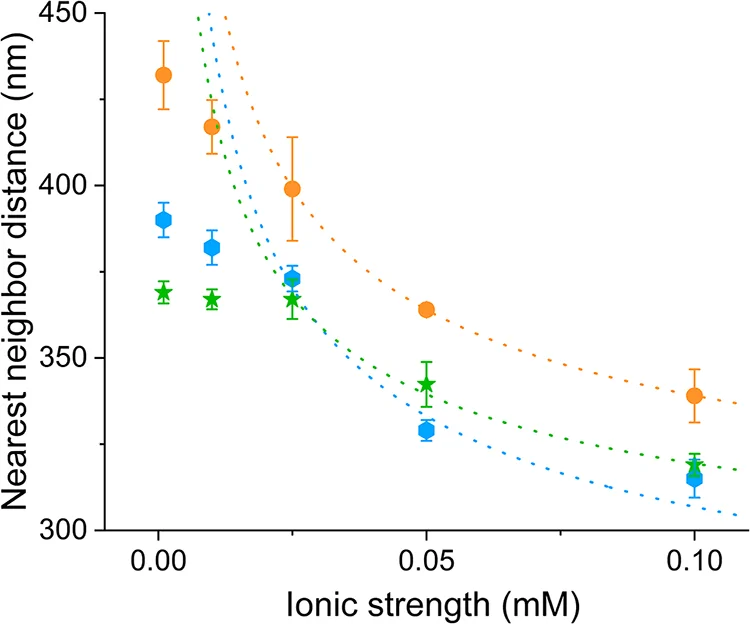
Average nearest-neighbor distances from three independent experiments are given as a function of ionic strengths of LaCl_3_ for vSP-2.1 (green stars), mSP-1.2 (blue hexagons), and sSP-0.3 (orange circles). The dotted lines represent the fit functions.

As we demonstrated in our previous research on medium-rough particles and higher ionic strengths (0.05 mM to 6 mM),^38,44^ the two-dimensional particle ordering is governed by the electrostatic field in the vicinity of the particle surface. Therefore, in these cases, the interparticle distance scales with the Debye length (κ^–1^), which is proportional to the inverse square root of the ionic strength (I), according to the following eq 1

1 $$\kappa^{-1}=\sqrt{\frac{k_{B}T\varepsilon_{0}\varepsilon }{2q^{2}N_{A}I}}$$

where *k*_B_ is the Boltzmann constant, *T* is the temperature, ε_0_ is the permittivity of vacuum, ε is the dielectric constant of the medium, *q* is the charge of the electron, and *N*_A_ is Avogadro’s constant.

Based on this, we examined whether similar behavior persists as particle roughness increases and ionic strength decreases. A plot of the nearest-neighbor distance (NND) versus *I* ^–1/2^ shows an approximately linear relationship for the three lowest *I* ^–1/2^ values, consistent with our earlier results^38,44^ (see Figure S4 in the Supporting Information). However, for *I* ^–1/2^ = 10 mM^–1/2^, the NND values for all three particle types deviate from this trend; these data points were therefore excluded from the linear fits. The data obtained in ultrapure water, corresponding to *I* ≈ 0.001 mM, were also excluded from this analysis because the ionic strength is extremely low and cannot be determined with sufficient accuracy (see the discussion of the zeta potential above and in the Supporting Information).

Accordingly, the data in Figure 6 are fitted using the corresponding nonlinear fitting function (eq 2).

2 $$NND=A+B\times I^{-1/2}$$

where NND is the nearest neighbor distance, and *A* and *B* are fitting parameters. For all three particle types, the data at the three highest ionic strengths are well described by eq 2, as observed earlier at higher ionic strengths. At the two lowest ionic strengths, however, the NND either remains essentially constant for vSP-2.1 or increases less strongly for the smoother particle types, mSP-1.2 and sSP-0.3. Therefore, the nonlinear fits were restricted to the three highest ionic strength values. Therefore, the nonlinear fits were restricted to the three highest ionic strength values. In contrast to the plot shown as a function of *I* ^–1/2^ in Figure S4, the data points at the lowest ionic strength (*I* ≈ 0.001 mM) were included in Figure 6, where the data are plotted against *I*. Thus, the uncertainty associated with the lowest ionic strength has only a minor effect on the displayed values.

The analyses in Figure S4 and Figure 6 indicate that, at low ionic strengths, particle roughness increasingly governs the ordering process rather than electrostatic interactions alone. Across the investigated ionic strength range, the NND decreased by approximately 22 %, 19 %, and 13 % for sSP-0.3, mSP-1.2, and vSP-2.1, respectively. This trend suggests that the dependence of interparticle distance on the ionic strength decreases as the particle roughness increases. When the particle roughness is sufficiently high, as in the case of vSP-2.1, the ordering process even appears to become independent of the ionic strength at low values. This result nicely demonstrates the effect of particle roughness on colloidal ordering on planar surfaces.

#### 3.2.3 Arrangement of Amino-Functionalized Silica Particles from Water on a Smooth Substrate

To examine the effect of substrate roughness, we investigated the particle arrangement on smooth gold substrates with an *R*_q_ of 0.57 ± 0.04 nm, as measured by AFM, and compared it with that on rough gold substrates (*R*_q_ = 1.62 ± 0.02 nm, see Section 3.2.2). Figure 7 shows FESEM images of NCPS prepared from vSP-2.1, mSP-1.2, and sSP-0.3 assembled on these substrates, along with the corresponding histograms. In the structures prepared on smooth substrates, the average nearest-neighbor distances of vSP-2.1 and mSP-1.2, calculated excluding aggregated particles, decrease uniformly by 9 % compared to those measured on the rougher substrates (see above), whereas a 20 % reduction was observed for the smoother particles (sample sSP-0.3). This decrease in nearest-neighbor distances on the smooth substrates is likely linked to differences in particle mobility after adsorption. The decrease in interparticle distances in all cases can be attributed to lower friction and weaker interlocking due to the smoother substrate. This behavior can be explained by a model proposed by Korayem et al., which simulates micro- and nanomanipulation, predicting that particles smaller than 500 nm tend to move by sliding on smooth surfaces.^68^ Rough particles, however, interlock, preventing this directional sliding and resulting in rolling motion.^65^ In a study by Fuchs et al., the increase in friction coefficients for rolling and sliding motions of 10 μm borosilicate microspheres on substrates with increasing root-mean-square roughness from 0.3 to 2.7 nm was measured using a nanoindenter setup.^69^ This effect is particularly pronounced for the smooth particles, suggesting that such interactions are significantly weakened when neither the substrate nor the particles have surface structures that enable substantial interlocking. Such conditions could allow more extensive lateral rearrangement during assembly and hence contribute to greater reduction in the nearest-neighbor distance compared to the rougher particles. Notably, for the two rougher particle types, the interparticle distance decreases to the same extent. This suggests that even a moderate degree of particle roughness can be sufficient to influence the balance of attractive and repulsive interactions, thereby limiting closer particle approach.

**Figure 7:**
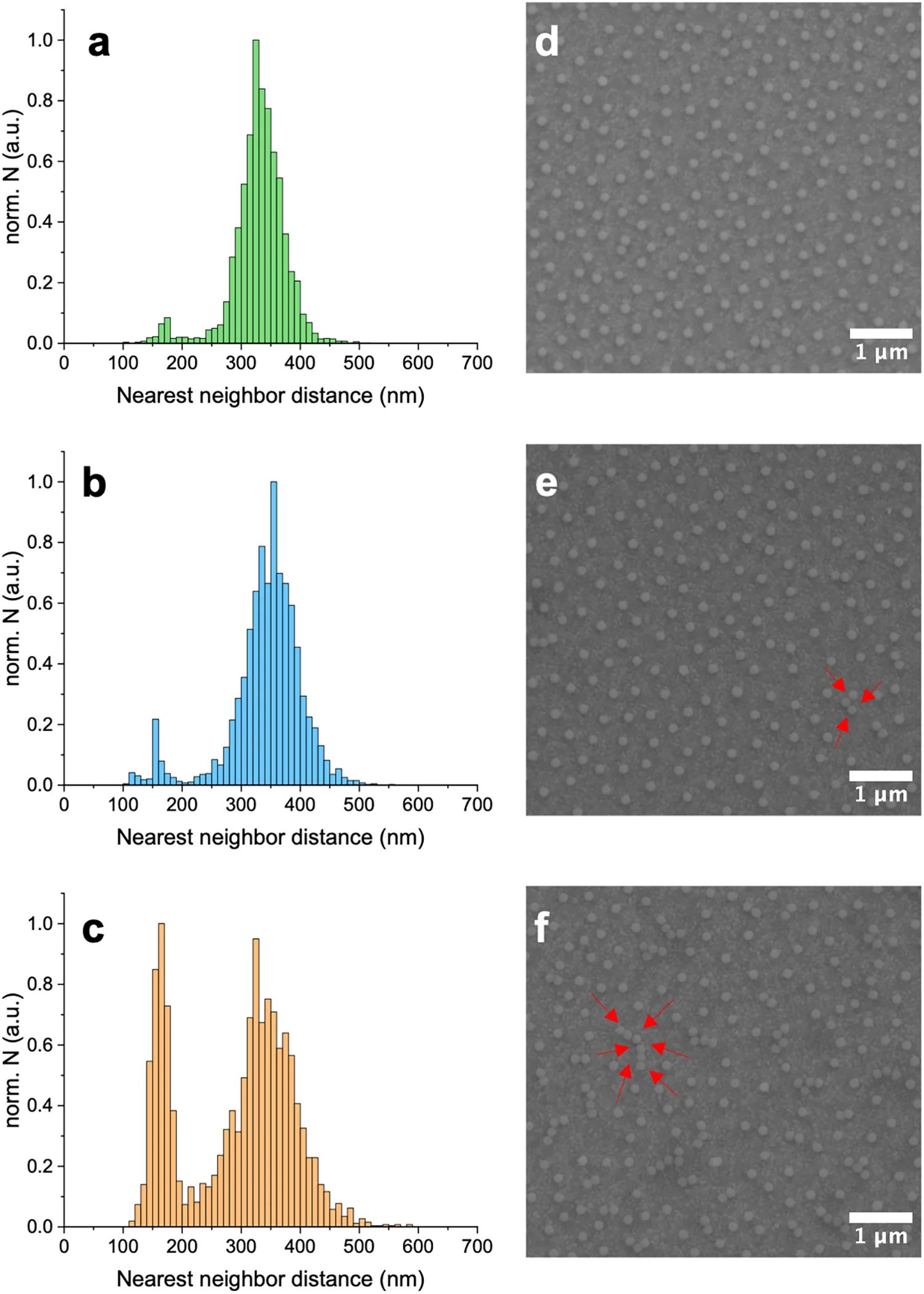
FESEM images and the corresponding histograms demonstrating the distribution of nearest-neighbor distances of amino-functionalized (a, d) vSP-2.1, (b, e) mSP-1.2, and (c, f) sSP-0.3 dispersed in water and transferred onto smooth gold substrates.

However, noticeable differences in particle aggregation behavior were observed among all three particle types on smooth substrates. A comparative representation of the aggregation percentages, including the corresponding standard deviations, is shown in Figure S6 in the Supporting Information. The percentage of aggregates and the extent to which they increase compared to the rougher substrates are higher in the NCPS of mSP-1.2 and sSP-0.3. For mSP-1.2, the percentage increases from 0.7 % to 4.5 % (see Figures 5b and 7b), and for sSP-0.3, it increases from 6.5 % to 22 % (see Figures 5c and 7c). In contrast, the aggregation percentage in the NCPS prepared from vSP-2.1 shows only a slight increase, from 1.9 % on the rough substrate to 2.5 % on the smooth substrate (see Figures 5a and 7a). The pronounced surface roughness of vSP-2.1 may lead to strong frictional interactions with the substrate, potentially hindering lateral particle mobility and thereby reducing its sensitivity to changes in substrate roughness. Upon careful examination of the FESEM images (at least six images at different spots on the substrate were acquired), revealed that NCPS prepared with mSP-1.2 form aggregates consisting of up to three particles, whereas those prepared with sSP-0.3 form aggregates of up to six particles (see Figure S5 in the Supporting Information). In the vicinity of these aggregates, areas on the substrate without adsorbed particles are found, indicating particle movement after random adsorption (see Figure 7). Smooth particles exhibit high mobility, and the hindrance of their movement is so low that aggregation occurs more frequently. Even aggregates consisting of many particles were observed, a phenomenon that had not been observed in our previous studies, even at much higher ionic strengths (3 mM) than in the present work, except in the presence of the most destabilizing salts such as NaNO_3_ and C­(NH_2_)_3_Cl.^38–40^ This finding highlights the importance of roughness for the formation of ordered structures. A key observation is the pronounced difference between medium-rough and virus-like particles. Their behavior differs little at larger distances, as shown by their average nearest-neighbor distances. However, differences in the varying degrees of particle roughness become more important at short distances, i.e., during aggregate formation, when stronger attractive forces act on the particles.

Surface roughness modifies interparticle interactions as particles approach one another; this becomes especially important at shorter distances close to those at which aggregation occurs. In this range, van der Waals forces strongly influence interparticle interactions, and they are also strongly influenced by surface asperities of the same size.^70^ In the case of rougher particles, the van der Waals forces at the point of first mechanical contact between two particles decrease with increasing roughness, compared to smoother particles, while the rest of the particle surfaces are more separated than those of the smoother particles. Consequently, attractive van der Waals forces are weaker between rough particles.^70^ In line with this, it has been experimentally shown that aggregation between rough particles due to attractive van der Waals forces is less likely than between smooth particles of the same material.^71,72^

Moreover, van der Waals interactions between colloid particles are short-ranged and decay rapidly with increasing interparticle distances.^22^ Thus, they become more dominant as the distance between particles decreases. This may explain why small differences in frictional forces, associated with roughness-induced mechanical hindrance, become more important as the interparticle distance decreases, potentially counteracting van der Waals attraction. Accordingly, differences in the surface roughness of particle types vSP-2.1 and mSP-1.2 may contribute to their distinct aggregation behavior, whereas they are expected to have a much lower effect on long-range electrostatic interactions.

## 4 Conclusions

Surface roughness modulates the particle arrangement on a substrate; therefore, effective control of colloidal self-assembly requires regulation of both particle and substrate roughness. Here, we examined the influence of particle and substrate roughness on particle arrangements in non-close-packed structures (NCPS). Silica particles with well-controlled size, low polydispersity, and systematically tunable roughness were synthesized in three morphologies: smooth, medium-rough, and rough with a virus-like surface structure. The synthesis of virus-like particles was optimized to achieve a wide roughness range (1.4–5.1 nm) and ∼5% polydispersity in a selected size range.

Amino-functionalized model silica particles with smooth (*R*_q_ = 0.3 nm) and medium-rough (*R*_q_ = 1.2 nm) surface morphologies, as well as rough virus-like particles with three different levels of roughness (*R*_q_ = 2.1, 4.3, and 5 nm), were assembled onto smooth and rough gold substrates (*R*_q_ = 0.57 and 1.62 nm), forming NCPS. The two roughest virus-like particles form pronounced, three-dimensional aggregates on rough substrates, likely due to roughness-driven mechanical interlocking, already in dispersion, as evidenced by dynamic light scattering. To investigate the impact of surface roughness on particle ordering as interparticle forces increase, the ionic strength of the dispersion medium was varied between 0.01 mM and 0.1 mM. Increasing the ionic strength enhances electrostatic screening, shortens the Debye length, and can reduce interparticle distances. However, on the rough substrates, the least-rough virus-like particles (*R*_q_ = 2.1 nm) showed no measurable change in nearest-neighbor distance across the three lowest ionic strengths (0.01–0.025 mM), while the smooth (*R*_q_ = 0.3 nm) and medium-rough particles (*R*_q_ = 1.2 nm) were affected by this change. Also, the overall decrease in nearest-neighbor distance was smaller for the virus-like particles. This reduced sensitivity to ionic strength suggests that surface asperities may weaken the influence of capillary interactions on changes in interparticle distances.

A comparison of NCPS formation from aqueous dispersions on two substrates with different roughnesses is consistent with greater lateral motion on the smoother substrate, leading to closer packing. This effect was the strongest for smooth particles, possibly reflecting lower friction and weaker interlocking. On the contrary, the two rougher particle types showed similar decreases in interparticle distances, suggesting that even a moderate roughness may be sufficient to limit rearrangement significantly. Notably, the aggregation percentage for virus-like particles (*R*_q_ = 2.1 nm) remains almost the same on both substrate types, whereas it significantly increases for the smooth and medium-rough particles. This behavior may be related to the strong distance-dependence of van der Waals forces, which become dominant at small interparticle distances. The present results suggest that roughness-induced friction may play a key role in hindering close particle contact. Thus, differences in surface roughness significantly modulate aggregation behavior. Overall, these findings demonstrate that the formation of non-close-packed ordered colloidal structures on surfaces can be well controlled by particle and substrate roughness, especially if the resulting effects are further tuned by varying the electrostatic interaction between particles on the substrate, suggesting that adjusting surface roughness provides control over particle arrangement. This opens new opportunities for the controlled formation of non-close-packed colloidal arrays and provides a straightforward tool for studying the effects of roughness on colloidal interactions in detail.

## Supplementary Material



## Notes

Additional details on the synthesis conditions of virus-like silica particles and corresponding particle size and polydispersity; additional TEM and FESEM images of silica particles with different surface roughnesses; DLS, electrophoretic mobility, and zeta potential data of amino-functionalized silica particles; nearest-neighbor distance distributions at different ionic strengths and their dependence on the inverse square root of ionic strength; FESEM images showing the ordering and aggregates of amino-functionalized silica particles with two different surface roughnesses on smooth gold substrates; and aggregation percentages of amino-functionalized silica particles with different surface roughnesses on smooth and rough gold substrates (PDF)
