# Supplementary material for: Using the Interplay of Particle and Substrate Roughness to Modulate Particle Arrangements in Non-Close-Packed Colloidal Arrays
Source: ACS Omega. 2026 Oct 1;11(40):60941–55. doi: 10.1021/acsomega.6c04722 (PMC13670335; doi:10.1021/acsomega.6c04722)
Supplement: Supplementary file 1 [file ao6c04722_si_001.pdf]

Using the Interplay of Particle and Substrate Roughness to Modulate Particle Arrangements in Non-Close-Packed Colloidal Arrays

Melike Barak[†‡§], *Florian Wolf*[†§], *Daniel Nachtsheim*[†‡§], *and Christina Graf*[†§]*

AUTHOR ADDRESS

† Department of Chemical Engineering and Biotechnology, Darmstadt University of Applied Sciences, Stephanstr. 7, 64295 Darmstadt, Germany

‡ Eduard-Zintl-Institute of Inorganic and Physical Chemistry, Technical University of Darmstadt, Peter-Grünberg-Str. 12, 64287 Darmstadt, Germany

§ EUt+ Institute of Nanomaterials and Nanotechnologies, EUTINN, European University of Technology, European Union, https://www.univtech.eu/eutinn

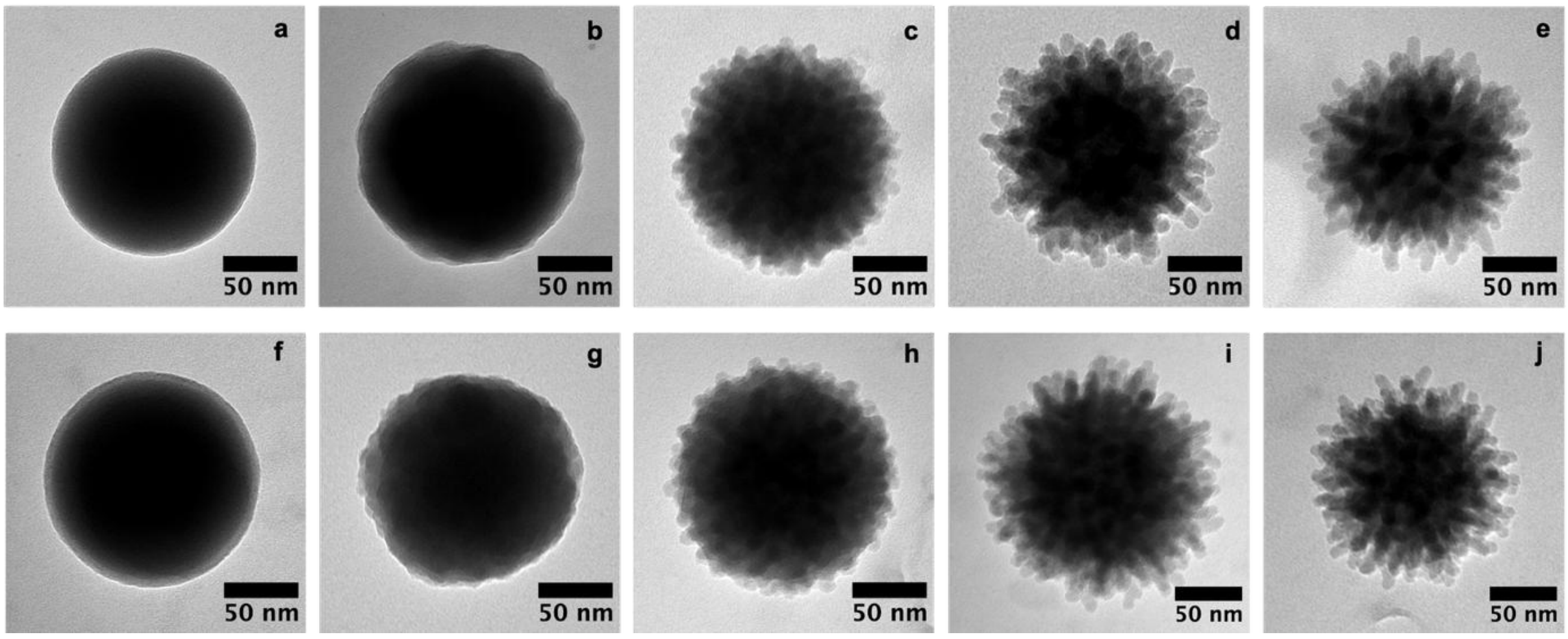

Figure S1. TEM images of (a) sSP ($R_q = 0.26 \pm 0.04$ nm), (b) mSP ($R_q = 1.0 \pm 0.3$ nm), (c) vSP-3 ($R_q = 2.5 \pm 0.2$ nm), (d) vSP-5 ($R_q = 4.5 \pm 0.3$ nm), and (e) vSP-22 ($R_q = 4.7 \pm 0.4$ nm) and amino-functionalized (f) sSP-0.3, (g) mSP-1.2, (h) vSP-2.1, (i) vSP-4.3, and (j) vSP-5.

Table S1. Synthesis conditions of vSP and corresponding particle size and polydispersity. FESEM and TEM images of the highlighted experiments are shown in **Fig. 2 and 3.**

Sample name	CTAB (M)	NaOH (mM)	Stirring rate (rpm)	Time (day)	Size (nm)	PD (%)
**vSP-1**	0.11	0.4	300	6	gel	-
**vSP-2**	0.096	1.2	300	6	204 ± 17	8
**vSP-3**	0.11	1.2	300	6	153 ± 7	5
**vSP-4**	0.08	1.2	300	6	147 ± 18	12
**vSP-5**	0.055	1.2	300	6	154 ± 12	8
**vSP-6**	0.055	1.6	300	6	115 ± 17	15
**vSP-7**	0.08	1.2	300	6	150 ± 15	10
**vSP-8**	0.055	0.8	300	6	160 ± 18	11
**vSP-9**	0.055	1.2	300	4	166 ± 13	8
**vSP-10**	0.11	1.2	300	6	126 ± 5	4
**vSP-11**	0.055	1.2	300	5	147 ± 11	7
**vSP-12**	0.069	1.2	300	6	147 ± 8	5
**vSP-13**	0.08	1.2	300	6	154 ± 8	5

Sample name	CTAB (M)	NaOH (mM)	Stirring rate (rpm)	Time (day)	Size (nm)	PD (%)
**vSP-14**	0.069	1.2	300	6	142 ± 7	5
**vSP-15**	0.08	1.2	300	6	128 ± 8	6
**vSP-16**	0.055	1.2	300	6	96 ± 9	9
**vSP-17**	0.069	1.2	300	6	119 ± 12	10
**vSP-18**	0.08	1.2	300	6	130 ± 11	8
**vSP-19**	0.069	1.2	300	6	117 ± 12	10
**vSP-20**	0.096	1.2	300	6	147 ± 11	7
**vSP-21**	0.11	1.2	300	4	121 ± 8	7
**vSP-22**	0.055	0.8	300	4	126 ± 8	6
**vSP-23**	0.055	1.2	300	4	109 ± 11	10
**vSP-24**	0.11	1.2	300	4	151 ± 7	5
**vSP-25**	0.055	1.2	300	4	104 ± 9	9
**vSP-26**	0.11	1.2	50	4	173 ± 6	3
**vSP-27**	0.055	1.2	50	4	170 ± 14	8
**vSP-28**	0.055	0.8	50	4	188 ± 19	10
**vSP-29**	0.11	1.2	300	4	108 ± 7	6
**vSP-30**	0.055	1.2	300	4	103 ± 9	9
**vSP-31**	0.055	1.6	300	4	101 ± 10	10
**vSP-32**	0.11	1.2	150	4	126 ± 8	6
**vSP-33**	0.055	0.8	150	4	136 ± 12	9
**vSP-34**	0.055	1.2	150	4	115 ± 10	9
**vSP-35**	0.055	1.6	150	4	110 ± 9	8
**vSP-36**	0.08	1.2	300	6	146 ± 12	8
**vSP-37**	0.096	1.2	300	6	157 ± 9	6
**vSP-38**	0.11	1.2	300	6	166 ± 9	5
**vSP-39**	0.096	1.2	300	6	154 ± 11	7
**vSP-40**	0.11	1.2	300	6	158 ± 9	6

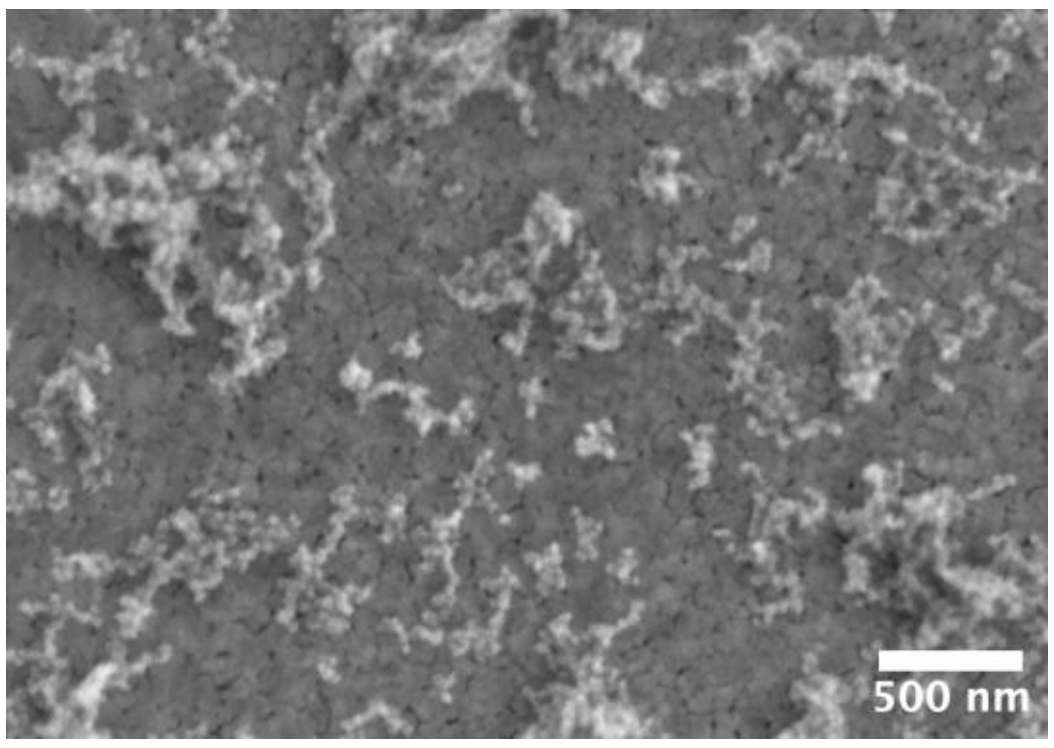

Figure S2. FESEM of vSP-1 prepared with 0.4 mM NaOH and 0.11 M CTAB at 6 days.

Table S2. DLS, electrophoretic mobility, and zeta potential data of amino-functionalized silica particles in ultrapure water and aqueous $LaCl_3$ solutions at ionic strengths ranging from 0.01 mM to 0.1 mM. Since the measurements were performed at very low ionic strengths, electrophoretic mobilities are reported as a primary, model-independent experimental parameter.[1] In ultrapure water, electrophoretic mobilities could be measured, although it is challenging to get a stable and homogeneous electric field under these conditions.[2] Based on Li et al.[3] and considering our experimental conditions (see Experimental Part), the ionic strength of the ultrapure water was assumed to be ~ 0.001 mM. Owing to the large Debye length, the system approaches the Hückel limit, and the Henry correction is negligible. The corresponding zeta potential values are therefore reported as apparent values. For the $LaCl_3$ solutions at ionic strengths of 0.01 and 0.1 mM, zeta potentials were corrected using Henry's equation.

**Ionic strength (mM)**	**vSP-2.1**			**mSP-1.2**			**sSP-0.3**		
	**D_{DLS} (nm)**	**Electrophoretic mobilities (µmcm/Vs)**	**Zeta potential (mV)**	**D_{DLS} (nm)**	**Electrophoretic mobilities (µmcm/Vs**	**Zeta potential (mV)**	**D_{DLS} (nm)**	**Electrophoretic mobilities (µmcm/Vs**	**Zeta potential (mV)**
**Ultrapure water**	178 ± 2	2.46 ± 0.12	32 ± 2	171 ± 3	3.51 ± 0.16	45 ± 2	180 ± 1	5.24 ± 0.13	67 ± 2
**0.01**	180 ± 2	3.96± 0.09	74 ± 2	167 ± 4	4.51 ± 0.11	85 ± 2	182 ± 1	3.23 ± 0.02	61 ± 1
**0.025**	174 ± 3	3.63± 0.10	67 ± 2	158 ± 5	4.51 ± 0.11	83 ± 1	180 ± 5	3.22 ± 0.06	60 ± 1
**0.05**	177 ± 6	2.96 ± 0.07	53 ± 1	169 ± 3	3.94 ± 0.05	71 ± 1	179 ± 3	3.24 ± 0.07	59 ± 1
**0.1**	177 ± 6	3.42 ± 0.06	60 ± 1	171 ± 2	3.75 ± 0.07	66 ± 1	174 ± 4	3.58 ± 0.03	63 ± 1

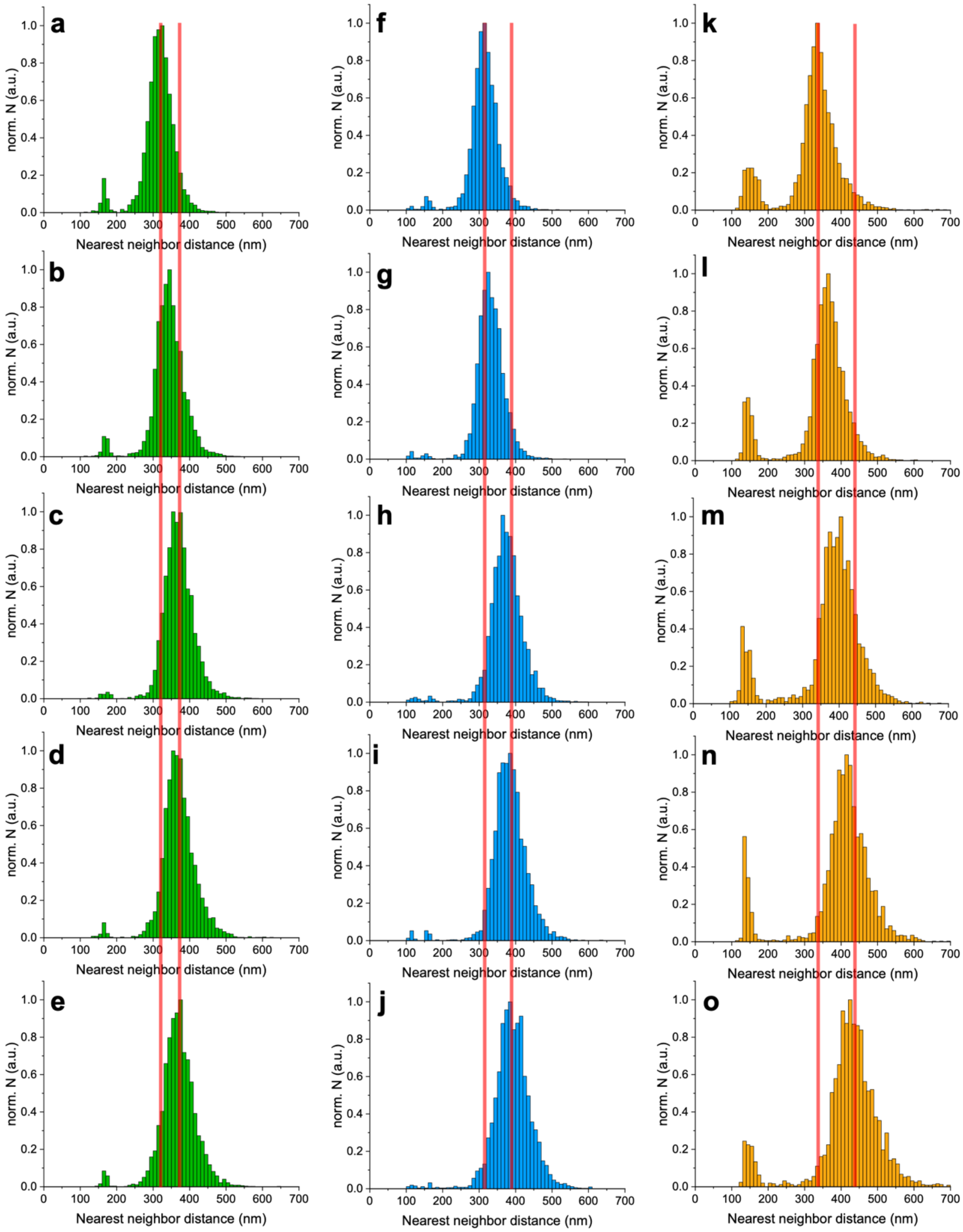

Figure S3. Histograms showing the distribution of the nearest-neighbor distances of NCPS of amino-functionalized silica particles with different roughnesses on gold substrates with R_q = 1.62 ± 0.02 nm. In green: histograms of NCPS prepared from highly rough virus-like silica particles (vSP-2.1): dispersed in aqueous $LaCl_3$ solutions with ionic strengths of (a) 0.1 mM, (b) 0.05 mM, (c) 0.025 mM, (d) 0.01 mM, and (e) in water. In blue: histograms of NCPS

prepared from medium-rough silica particles (mSP-1.2) dispersed in aqueous $LaCl_3$ solutions with ionic strength of (f) 0.1 mM, (g) 0.05 mM, (h) 0.025 mM, (i) 0.01 mM, and (j) in water, and in orange: histograms of NCPS prepared from smooth silica particles (sSP-0.3) dispersed in aqueous $LaCl_3$ solutions with ionic strength of (k) 0.1 mM, (l) 0.05 mM, (m) 0.025 mM, (n) 0.01 mM, and (o) in water. The red lines indicate the average nearest-neighbor distance at the highest and lowest ionic strength values for each class of nanoparticles.

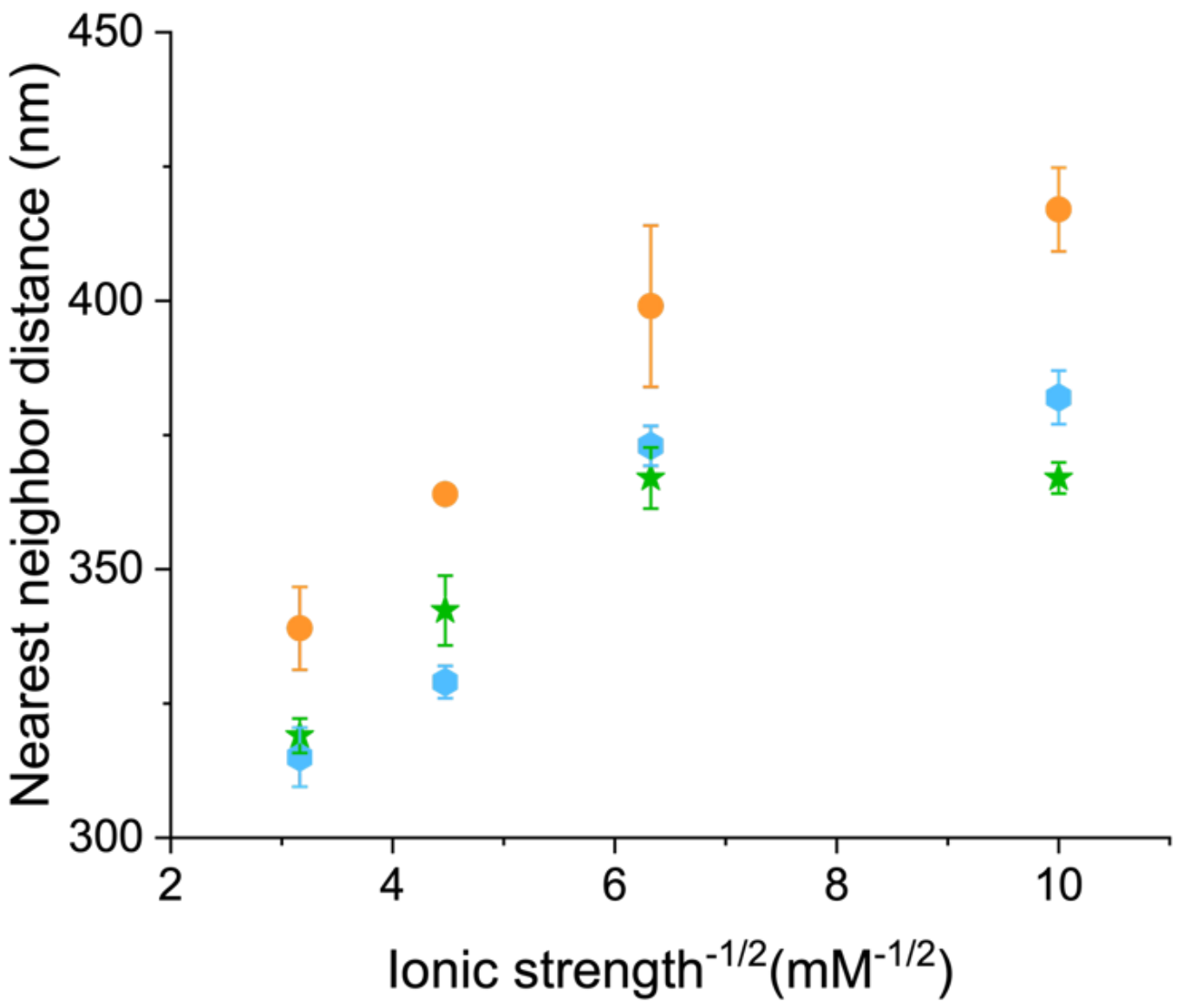

Figure S4. The average nearest-neighbor distances from three independent experiments are given as a function of the inverse square root of ionic strengths for vSP-2.1 (green stars), mSP-1.2 (blue hexagons), and sSP-0.3 (orange circles).

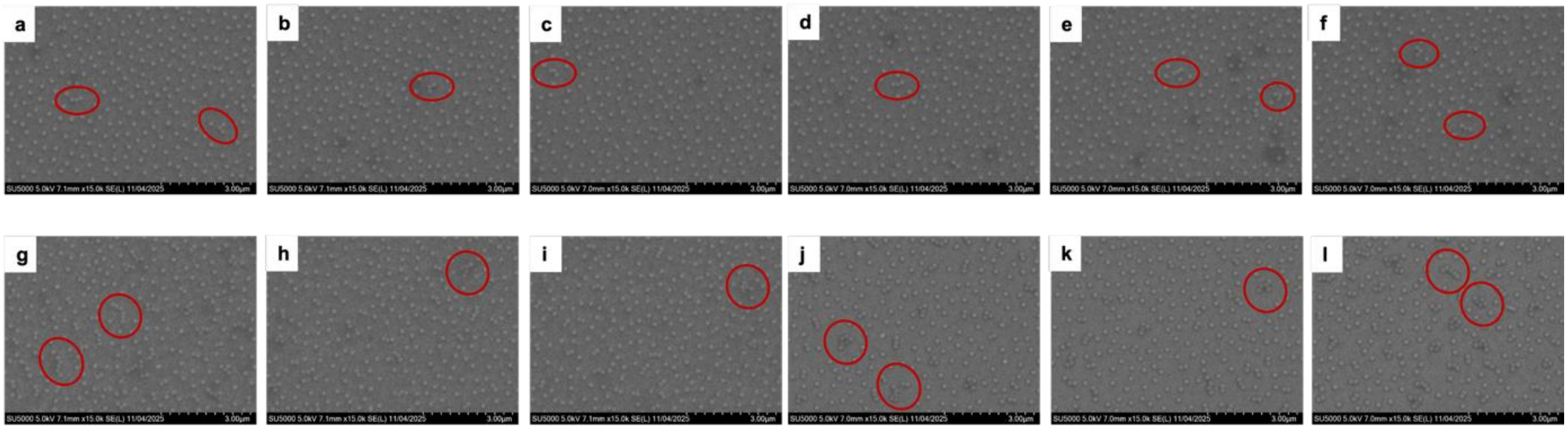

Figure S5. FESEM images of amino-functionalized (a-f) mSP-1.2 and (g-l) sSP-0.3 dispersed in water and transferred onto smooth gold substrates with marked aggregates.

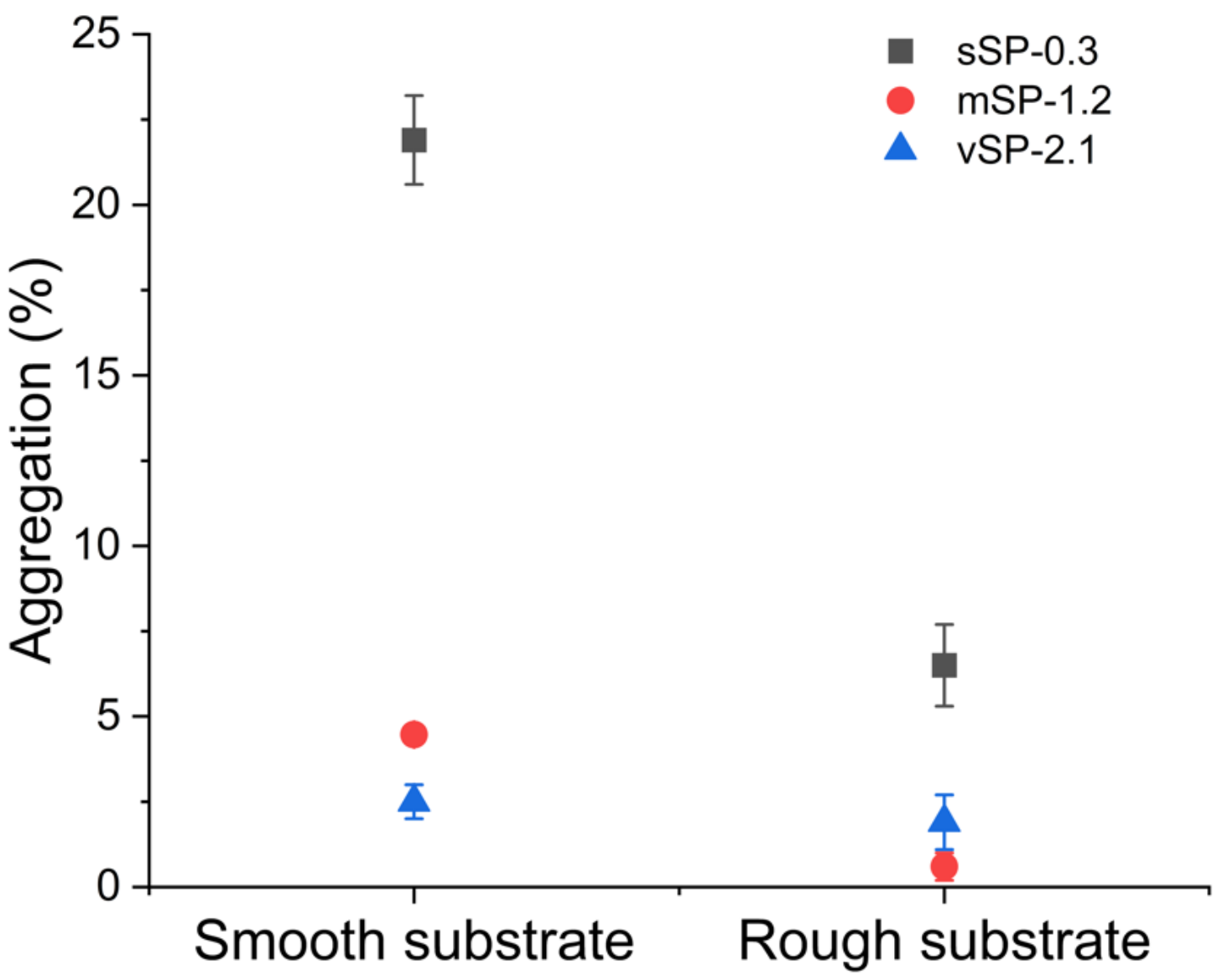

Figure S6. Aggregation percentages of amino-functionalized silica particles with different roughnesses on smooth and rough gold substrates.

References

(1) Skoglund, S.; Hedberg, J.; Yunda, E.; Godymchuk, A.; Blomberg, E.; Wallinder, I. O. Difficulties and Flaws in Performing Accurate Determinations of Zeta Potentials of Metal Nanoparticles in Complex Solutions—Four Case Studies. *PLOS ONE* **2017**, *12* (7), e0181735. https://doi.org/10.1371/journal.pone.0181735.

(2) *Zeta potential method development*. https://www.malvernpanalytical.com/en/learn/knowledge-center/technical-notes/tn220915-zeta-potential-method-development (accessed 2026-08-27).

(3) Li, X.; Batchelor-McAuley, C.; Laborda, E.; Compton, R. G. Aqueous Voltammetry in the Near Absence of Electrolyte. *Chem. – Eur. J.* **2017**, *23* (60), 15222–15226. https://doi.org/10.1002/chem.201703525.
